# Association of antihypertensive drugs with fracture and bone mineral density: A comprehensive drug-target Mendelian randomization study

**DOI:** 10.3389/fendo.2023.1164387

**Published:** 2023-03-28

**Authors:** Xin Huang, Tianxin Zhang, Ping Guo, Weiming Gong, Hengchao Zhu, Meng Zhao, Zhongshang Yuan

**Affiliations:** ^1^ Department of Biostatistics, School of Public Health, Cheeloo College of Medicine, Shandong University, Jinan, Shandong, China; ^2^ Institute for Medical Dataology, Shandong University, Jinan, Shandong, China; ^3^ Department of Biostatistics, School of Public Health, Yale University, New Haven, CT, United States; ^4^ Department of Endocrinology, Shandong Provincial Hospital Affiliated to Shandong First Medical University, Jinan, Shandong, China

**Keywords:** antihypertensive drugs, fracture, bone mineral density, drug-target mendelian randomization, causal effect

## Abstract

**Background:**

Observational studies have investigated the associations between antihypertensive drugs and fracture risk as well as bone mineral density (BMD), but yielding controversial results.

**Methods:**

In this study, a comprehensive drug-target Mendelian randomization (MR) analysis was conducted to systematically examine the associations between genetic proxies for eight common antihypertensive drugs and three bone health-related traits (fracture, total body BMD [TB-BMD], and estimated heel BMD [eBMD]). The main analysis used the inverse-variance weighted (IVW) method to estimate the causal effect. Multiple MR methods were also employed to test the robustness of the results.

**Results:**

The genetic proxies for angiotensin receptor blockers (ARBs) were associated with a reduced risk of fracture (odds ratio [OR] = 0.67, 95% confidence interval [CI]: 0.54 to 0.84; *P* = 4.42 × 10^-4^; *P-*adjusted = 0.004), higher TB-BMD (β = 0.36, 95% CI: 0.11 to 0.61; *P* = 0.005; *P-*adjusted = 0.022), and higher eBMD (β = 0.30, 95% CI: 0.21 to 0.38; *P* = 3.59 × 10^-12^; *P-*adjusted = 6.55 × 10^-11^). Meanwhile, genetic proxies for calcium channel blockers (CCBs) were associated with an increased risk of fracture (OR = 1.07, 95% CI: 1.03 to 1.12; *P* = 0.002; *P-*adjusted = 0.013). Genetic proxies for potassium sparing diuretics (PSDs) showed negative associations with TB-BMD (β = -0.61, 95% CI: -0.88 to -0.33; *P* = 1.55 × 10^-5^; *P-*adjusted = 1.86 × 10^-4^). Genetic proxies for thiazide diuretics had positive associations with eBMD (β = 0.11, 95% CI: 0.03 to 0.18; *P* = 0.006; *P-*adjusted = 0.022). No significant heterogeneity or pleiotropy was identified. The results were consistent across different MR methods.

**Conclusions:**

These findings suggest that genetic proxies for ARBs and thiazide diuretics may have a protective effect on bone health, while genetic proxies for CCBs and PSDs may have a negative effect.

## 1 Introduction

Blood pressure lowering through antihypertensive drugs is an established strategy for hypertension management to reduce the risk of developing cardiovascular diseases (1, 2). Hypertension and osteoporosis are often co-existed, especially in the aging population (3). The effects on osteoporosis from antihypertensive drugs can differ due to the diverse mechanisms of action among different classes of these drugs. The use of antihypertensive drugs should take the possible impact on bone health into account, so that the antihypertensive drugs benefiting bone health can be preferred.

The observational associations between antihypertensive drugs and fracture have been extensively studied, with the association direction and strength expected to be depended on the specific type of antihypertensive drug class. The cohort study in the Osteoporotic Fracture in Men Study (MrOS) reported that using angiotensin receptor blockers (ARBs) for prolonged periods reduced the risk of non-vertebral fractures in the elderly compared to using angiotensin-converting enzyme inhibitors (ACEIs) or calcium channel blockers (CCBs) (4). A recent meta-analysis illustrated that thiazide diuretics, beta-blockers (BBs), and ARBs could reduce the risk of hip fracture (5). Recent studies regarding the risk of falls in older adults have also raised concerns about hip fracture resulted from antihypertensive drug use (6, 7). However, the associations between different antihypertensive drugs and the occurrence of fracture remains largely inconsistent and controversial. These inconsistent findings may be attributed to differences in study populations and outcome definitions as well as bias due to unmeasured confounding. The observational design was commonly acknowledged to be insufficient to prove causality. Although randomized clinical trials (RCTs) are often regarded as the gold standard for determining causality, they can have limitations in terms of feasibility, cost, and ethics (8).

Mendelian randomization analysis has been developed as a powerful statistical method for assessing causal associations using genetic variables as instruments (9). Random assignment of genetic alleles effectively eliminates the effect of unobserved confounding factors and reduces measurement error, similar to randomization in RCTs (10). Given the wide availability of summary data from genome-wide association study (GWAS), MR could efficiently and economically assess the causal relationship between exposure and disease outcome (11). Drug-target MR can be used to predict drug development and repurposing opportunities by using genetic instruments in or near target genes to simulate the potential actions of drug targets (12).

In the present study, using single-nucleotide polymorphisms (SNPs) in or near drug target genes as drug genetic proxies, we conducted a comprehensive drug-target MR analysis systematically examining the causal associations between antihypertensive drug classes and bone health-related traits, including fractures, total body bone mineral density (TB-BMD), and estimated heel BMD (eBMD). The results can provide a helpful guide regarding the use of antihypertensive drugs, especially for those hypertension patients with poor bone health. In addition, the findings may promote the repurposing of antihypertensive drugs as a potential osteoporosis prevention strategy for future trial design.

## 2 Methods

### 2.1 Study design

**Figure 1:**
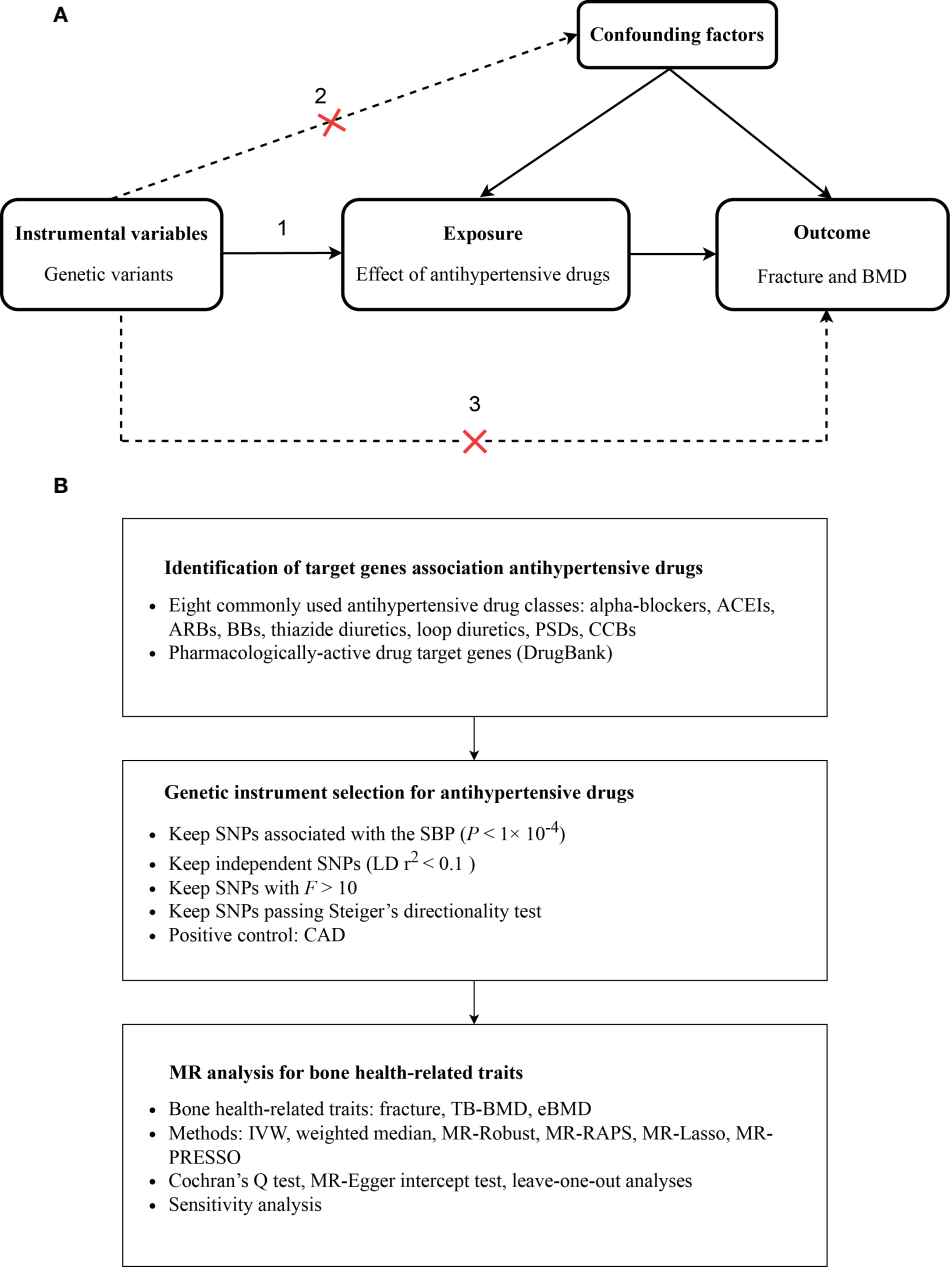
Diagram of the study design. **(A)** The drug-target MR framework in this study. Three assumptions are often required: (1) the selected instrument is predictive of the exposure, (2) the instrument is independent of confounding factors, and (3) there is no horizontal pleiotropy (the instrument is associated with the outcome only through the exposure). **(B)** The overall study design. IVW, inverse-variance weighted; MR, Mendelian randomization; MR-RAPS, Robust Adjusted Profile Score; MR-PRESSO, Mendelian randomization Pleiotropy RESidual Sum and Outlier; CAD, coronary artery disease; TB-BMD, total body bone mineral density; eBMD, estimated heel bone mineral density; ACEIs, angiotensin-converting enzyme inhibitors; ARBs, angiotensin receptor blockers; BBs, beta-blockers; PSDs, potassium sparing diuretics; CCBs, calcium channel blockers.

We estimated the causal associations of genetic proxies for antihypertensive drugs with bone health-related traits by a drug-target MR method, with the study design provided in **Figure 1**. We totally included eight commonly used antihypertensive drugs following the guidelines of the European Society of Cardiology/European Society of Hypertension (ESC/ESH) 2018 (13), including alpha-blockers, ACEIs, ARBs, BBs, thiazide diuretics, loop diuretics, potassium sparing diuretics (PSDs), and CCBs. Specifically, we first identified genetic variants associated with blood pressure (BP) lowering in drug target genes to proxy drug target effects. Then, the causal effects of these drug genetic proxies on fracture, TB-BMD, and eBMD were evaluated using multiple MR methods. A completed Study to Enhance the Use of Mendelian Randomization for Observational Epidemiology (STROBE-MR) statement was provided (14) (**Supplementary STROBE-MR checklist**).

### 2.2 Data source

Data sources were detailed in **Table 1**. All the GWAS summary data were with European ancestry. The GWAS summary statistics of bone health-related traits were obtained from the GEnetic Factors for Osteoporosis Consortium website (GEFOS, http://www.gefos.org/). Studies with sample sizes greater than 50,000 in the GEFOS consortium were selected to improve statistical power, including fracture, TB-BMD, and eBMD. The GWAS summary data for both fracture (53,184 cases and 373,611 controls) and eBMD (n = 426,824) were obtained from UKB with adjustment for sex, age, with the largest sample size to date (15). Fracture was defined using ICD-10 diagnostic codes, where the codes for skull, face, hand and foot fractures, pathological fractures caused by malignant tumors, atypical femoral fractures, periprosthetic fractures and healed fractures were excluded. Summary-level data for eBMD was measured using quantitative ultrasound. The summary data of TB-BMD, were provided by a meta-analysis (n = 56,284), with adjustment for age, weight, height, and measured utilizing dual-energy X-ray absorptiometry (DXA) (16).

**Table 1:** Summary of GWAS datasets included in this study.

Phenotype	Sample size (case/control)	Number of SNPs	Population	Adjustments^a^
Exposure				
SBP	757,601	7,088,083	European	Age, age^2^, sex, BMI
Outcomes				
Fracture	416,795(53,184/373,611)	13,977,204	European	Age, sex
eBMD	426,824	13,681,377	European	Age, sex
TB-BMD	56,284	16,162,733	European	Age, weight
CAD	547,261(122,733/424,528)	7,934,254	European	Age, sex

GWAS, genome-wide association study; SNPs, single nucleotide polymorphisms; eBMD, estimated heel bone mineral density; TB-BMD, total body bone mineral density; SBP, systolic blood pressure; CAD, coronary artery disease; BMI, body mass index.

a All GWAS studies have further adjusted for principal components.

The GWAS summary data for systolic blood pressure (SBP) was from a meta-analysis of the International Consortium for Blood Pressure (ICBP) (17). The GWAS was conducted using linear regression and combined across studies using inverse-variance weighted meta-analysis, including up to 757,601 participants with adjustment for age, age^2^, sex, and BMI. The GWAS summary data of coronary artery disease (CAD) (122,733 cases and 424,528 controls), which we included here as a positive control to validate genetic instruments, was obtained from CARDIoGRAMplusC4D, with adjustment for sex and age (18).

### 2.3 Genetic instrument selection for antihypertensive drugs

We selected SNPs associated with blood pressure (BP) lowering in or near drug target genes as proxies for different antihypertensive drugs. Specifically, we identified eight commonly used antihypertensive drug classes and collected information on the protein targets of these drugs from the DrugBank (19). We, following previous studies (20), selected SNPs in or near (± 200 kb) corresponding target genes as proxies for antihypertensive drug classes, if they are associated with SBP based on *P* < 1 × 10^-4^.

In addition, we estimated the F statistics of every instrument and only kept those variants with F > 10 to avoid weak instrumental bias (21). We then clumped these SNPs according to a lenient linkage disequilibrium (LD) threshold of r^2^ < 0.1 using the 1000 Genomes Project European reference panel. We employed MR Steiger filtering to exclude those genetic instruments which explained less variance on outcome than on exposure. Steiger filtering can infer the causal direction for an identified SNP between exposure and outcome through estimating and comparing the proportion of variance explained in each (22). To validate the genetic proxies for antihypertensive drug instruments, we further examined the associations between genetically proxied antihypertensive drugs and CAD, and compared them with previous meta-analyses of clinical trials (23, 24), which can be used as positive control since antihypertensive drugs are known to have cardiovascular protective effects.

### 2.4 Statistical analysis

Prior to the MR analysis, summary data for the instruments on exposure and outcome were harmonized. When a SNP was missing for the outcome association, a proxy (r^2^ ≥ 0.8) was sought for it, and those SNPs without proxies were further removed. Palindromic SNPs with moderate minor allele frequency (> 0.4) were removed, as allele reversal could not be inferred. Inverse variance weighting (IVW) was performed as the main MR analysis to obtain the estimate of the causal association. In addition, multiple MR methods with different model assumptions were used to evaluate the robustness and reliability of our findings, including (1) weighted median method, which is consistent if at least 50% of the weight comes from valid instrumental variables (25); (2) IVW method using robust regression (MR-Robust), which can reduce the standard error of estimates (26); (3) MR Robust Adjusted Profile Score (MR-RAPS), which is robust to both systematic and idiosyncratic pleiotropy (27); (4) MR-Lasso, which uses penalization to identify the candidate instrumental SNPs (26); (5) Mendelian randomization Pleiotropy RESidual Sum and Outlier (MR-PRESSO) analysis, which makes causal inference as well as outlier detection (28).

We used the R packages “MendelianRandomization” (29), “mr.raps” (27), and “MRPRESSO” (28) to conduct MR analyses. All the reported Odds ratios (OR) or causal effect estimates were corresponding to per 5 mmHg lower SBP to make the effect comparable. For the main analysis using IVW method, we chose a false discovery rate (FDR) method for multiple testing correction with the significance threshold being FDR adjust *P* < 0.05, in recognition that the Bonferroni correction for multiple non-independent tests may be too stringent. The nominally significant threshold (*P* < 0.05) was used for alternative MR methods. The *P* values after FDR correction were described as *P-*adjusted.

### 2.5 Sensitivity analysis

The MR-Egger method was used to check for potential pleiotropy by testing for a significantly deviating MR-Egger intercept from zero (30). Heterogeneity was assessed using Cochran’s Q statistics. Leave-one-out (LOO) analysis was carried out to test whether any single SNP contributed powerfully to the causal effects. In addition, we used the Phenoscanner tool (31, 32) to check whether any of the selected SNPs (or their proxies, r^2^ > 0.8) were associated with other risk factors of bone health-related traits (such as body mass index, smoking, stroke) (33) at genome-wide significance as potential pleiotropy. We then removed the identified potentially pleiotropic SNPs for sensitivity analysis to determine the robustness of the results.

## 3 Results

### 3.1 Genetic instrument selection and validation

For eight different antihypertensive drug classes, we identified a total of 38 pharmacological target genes (**Supplementary Table 1**) and chose genetic instruments within or nearby drug targets genes as proxies. Totally, 120 SNPs were obtained following the strict instrument screening procedure described above, with details provided in **Figure 1**. All of these instrumental SNPs had F statistics of more than 10, which suggests that the weak instrument bias was not expected to affect the results. Briefly, there were 12 SNPs for alpha-blockers, 5 for ACEIs, 4 for ARBs, 20 for BBs, 4 for thiazide diuretics, 9 for loop diuretics, 2 for PSDs, 64 for CCBs (**Supplementary Table 2**). Positive control results for the effects of genetic proxies of antihypertensive drugs on CAD were consistent with results from clinical trials, indicating the validity of these instrumental SNPs (**Supplementary Figure 1**).

### 3.2 Drug-target MR analysis for fracture

**Figure 2:**
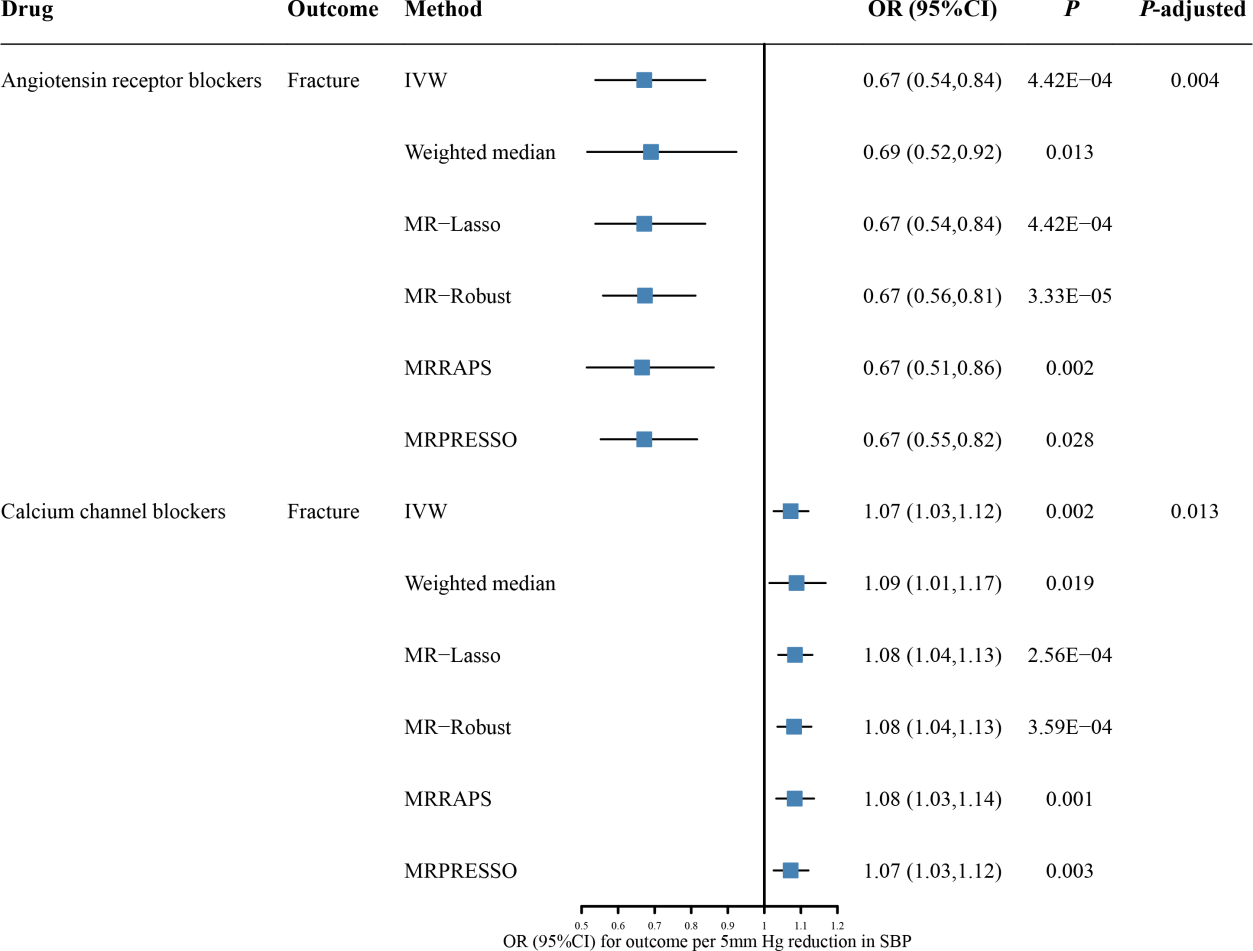
The causal effects of genetic proxies for antihypertensive drug classes on risk of fracture. CI, confidence interval; OR, odds ratio; SBP, systolic blood pressure; IVW, inverse-variance weighted; MR, Mendelian randomization; MR-RAPS, Robust Adjusted Profile Score; MR-PRESSO, Mendelian randomization Pleiotropy RESidual Sum and Outlier.

We first examined the causal effect of antihypertensive drugs on fracture risk using drug-target MR analysis (**Figure 2**; **Supplementary Table 4**). The genetic proxies for ARBs have shown a protective effect on fracture (OR = 0.67, 95% CI: 0.54 to 0.84; *P* = 4.42 × 10^-4^; *P-*adjusted = 3.53 × 10^-3^). The causal effect estimate was consistent from different MR methods. It was 0.69 (95% CI: 0.52 to 0.92; *P* = 0.013) in weighted median, 0.67 (95% CI: 0.54 to 0.84; *P* = 4.42 × 10^-4^) in MR-Lasso, 0.67 (95% CI: 0.56 to 0.81; *P* = 3.33 × 10^-5^) in MR-Robust, 0.67 (95% CI: 0.51 to 0.86; *P* = 0.002) in MR-RAPS, and 0.67 (95% CI: 0.55 to 0.82; *P* = 0.028) in MR-PRESSO. Genetically predicted CCBs were associated with higher fracture risk (OR = 1.07, 95% CI: 1.03 to 1.12; *P* = 0.002; *P*-adjusted = 0.013). Again, the results are consistent across different MR methods, including weighted median method (OR = 1.09, 95% CI: 1.01 to 1.17; *P* = 0.019), MR-Lasso (OR = 1.08, 95% CI: 1.04 to 1.13; *P* = 2.56 × 10^-4^), MR-Robust (OR = 1.08, 95% CI: 1.04 to 1.13; *P* = 3.59 × 10^-4^), MR-RAPS (OR = 1.08, 95% CI: 1.03 to 1.14; *P* = 0.001), and MR-PRESSO (OR = 1.07, 95% CI: 1.03 to 1.12; *P* = 0.003). In addition, further analysis suggested that no horizontal pleiotropy (Egger intercept *P* = 0.137 for ARBs and 0.157 for CCBs) and no heterogeneity (Cochran’Q test *P* = 0.947 for ARBs and 0.289 for CCBs) (**Supplementary Table 3**). The LOO plots showed no distortion, indicating that no single SNP can have a substantial impact on the results (**Supplementary Figure 2**).

### 3.3 Drug-target MR analysis for TB-BMD and eBMD

**Figure 3:**
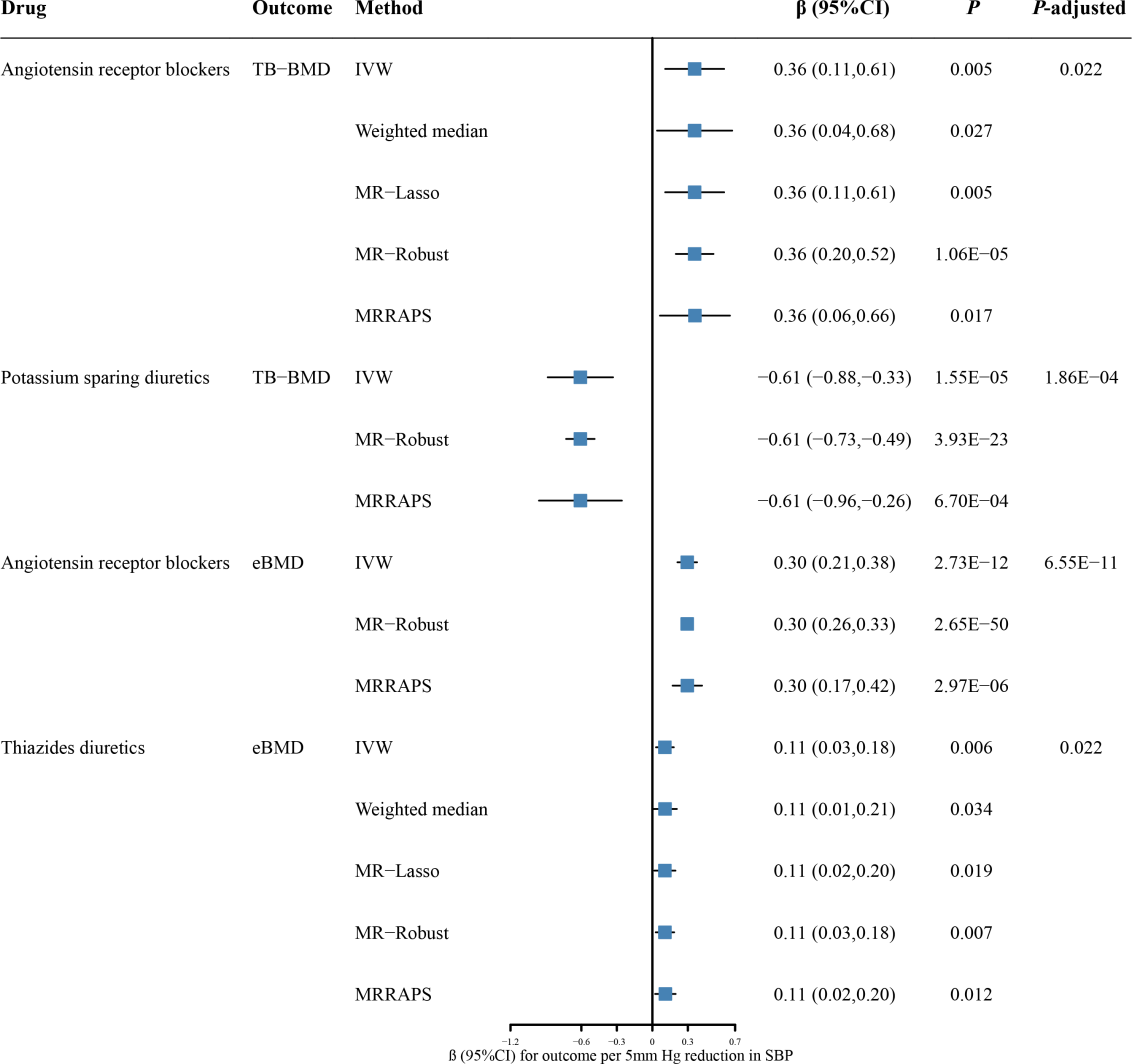
The causal effects of genetic proxies for antihypertensive drug classes on BMD. CI, confidence interval; TB-BMD, total body bone mineral density; eBMD, estimated heel bone mineral density; SBP, systolic blood pressure; IVW, inverse-variance weighted; MR, Mendelian randomization; MR-RAPS, Robust Adjusted Profile Score; MR-PRESSO, Mendelian randomization Pleiotropy RESidual Sum and Outlier.

The drug-target MR analysis was also performed to assess the causal effects of antihypertensive drugs on TB-BMD and eBMD (**Figure 3**; **Supplementary Tables 5, 6**). We identified significant causal associations of genetically predicted ARBs with higher TB-BMD (β = 0.36, 95% CI: 0.11 to 0.61; *P* = 0.005; *P*-adjusted = 0.022) (**Supplementary Table 5**). The results remained consistent across the weighted median (β = 0.36, 95% CI: 0.04 to 0.68; *P* = 0.027), MR-Lasso (β = 0.36, 95% CI: 0.11 to 0.61; *P* = 0.005), MR-Robust (β = 0.36, 95% CI: 0.20 to 0.52; *P* = 1.06 × 10^-5^), and MR-RAPS (β = 0.36, 95% CI: 0.06 to 0.66; *P* = 0.017). We also found associations between genetically predicted PSDs with higher TB-BMD (β = -0.61, 95% CI: -0.88 to -0.33; *P* = 0.039; *P*-adjusted = 1.86 × 10^-4^). However, the causal association should be interpreted cautiously given with only two SNPs for PSDs. There was no pleiotropy detected from MR-Egger regression model (MR-Egger intercept *P* = 0.514 for ARBs) or Cochran’s Q test (*P* = 0.880 for ARBs and 0.926 for PSDs) (**Supplementary Table 3**). The LOO analyses also showed no outlier SNPs (**Supplementary Figure 3**). In addition, genetic proxies for thiazide diuretics were positively associated with TB-BMD at a nominal threshold (β = 0.24, 95% CI: 0.01 to 0.47; *P* = 0.039; *P*-adjusted = 0.134) (**Supplementary Table 5**).

In a further assessment of antihypertensive drugs in eBMD, genetic proxies for ARBs and thiazide diuretics were associated with higher eBMD (β = 0.30, 95% CI: 0.21 to 0.38; *P* = 2.73 × 10^-12^; *P*-adjusted = 6.55 × 10^-11^ for ARBs; β = 0.11, 95% CI: 0.03 to 0.18; *P* = 0.006; *P*-adjusted = 0.022 for thiazide diuretics) (**Figure 3**; **Supplementary Table 6**). The causal effect estimates remained from the weighted median, MR-Lasso, MR-Robust, and MR-RAPS methods (**Figure 3**). Our analysis suggested no significant evidence of horizontal pleiotropy (Egger intercept *P* = 0.822 for thiazide diuretics). Furthermore, there was no statistically significant heterogeneity between antihypertensive drugs and eBMD (Cochran’Q test *P* = 0.646 for ARBs and 0.247 for thiazide diuretics) (**Supplementary Table 3**). The LOO analyses also showed no outlier SNPs (**Supplementary Figure 4**). There was no evidence of causal association for other antihypertensive drugs on eBMD (**Supplementary Table 6**).

### 3.4 Sensitivity analysis

There were six potentially pleiotropic SNPs identified by Phenoscanner (**Supplementary Table 7**), and after removing these SNPs, the results were consistent with previously significant associations (**Supplementary Table 8, 9**).

## 4 Discussion

**Figure 4:**
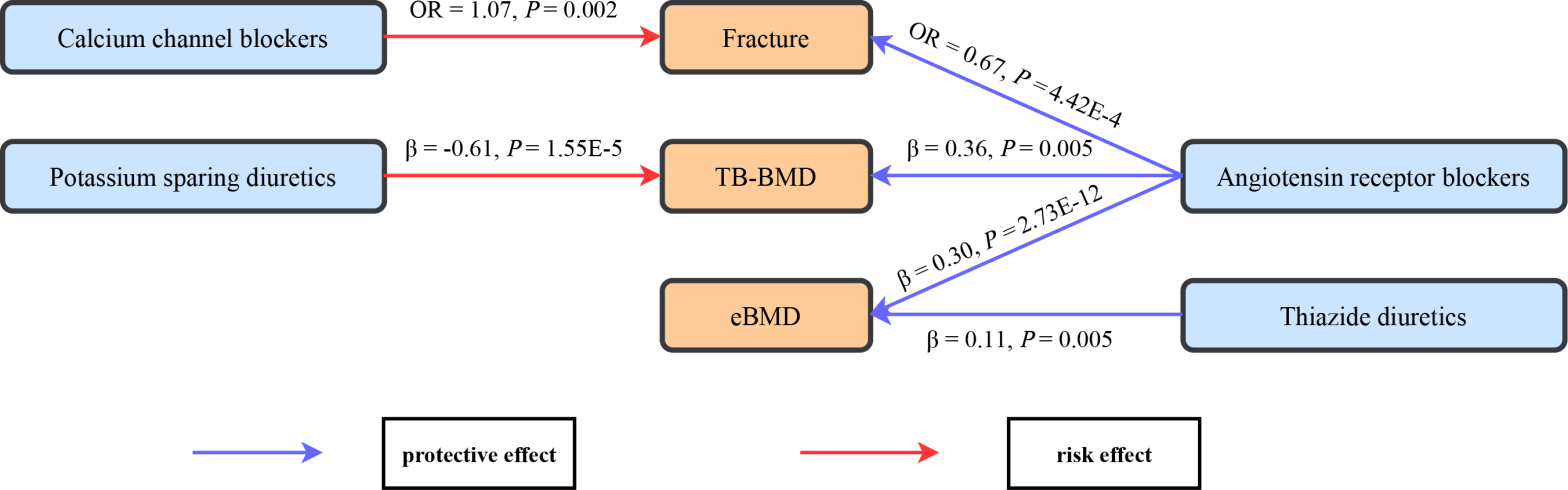
The schematic for the significant causal effects of antihypertensive drugs on bone health-related traits. Significant causal effects are presented as arrows with the estimated effect sizes alongside, with the red arrow representing risk effect and the blue arrow representing protective effects. TB-BMD, total body bone mineral density; eBMD, estimated heel bone mineral density.

A comprehensive drug target MR analysis was conducted to infer potential causal effects for antihypertensive drugs on fracture and BMD, with the schematic of the significant findings provided in **Figure 4**. The results showed that the genetic proxies for ARBs were associated with lower fracture risk, higher TB-BMD, and higher eBMD. Meanwhile, genetically predicted CCBs were associated with higher fracture risk. Genetic proxies for thiazide diuretics had positive associations with eBMD, while genetic proxies for PSDs showed negative associations with TB-BMD. More importantly, the results were verified by multiple MR methods, which suggested reliability and consistency.

Previous studies illustrated that ARBs use was associated with lower fracture risk compared to non-users. The study of the Women’s Health Initiative found that long-term ARBs use was associated with a reduced risk of all fractures in postmenopausal women (34). Another cohort study revealed a reduced risk of hip fracture in people with ARBs medications (35). Angiotensin II receptor blockers imposed their pharmacological effects by affecting the renin aldosterone angiotensin system (RAAS). In addition to acting systemically, RAAS played a role locally in bone tissue (36). Osteoblasts and osteoclasts have been discovered to express angiotensin II, indicating the presence of local RAAS in bone (37). RAAS might adjust bone metabolism *via* affecting the RANKL/RANK/OPG system (38). Thus, several animal studies have demonstrated the prevention of osteoporosis by blocking the angiotensin II pathway (39), increasing bone mass and strength (37) as well as accelerating bone healing and remodeling (40). Notably, consistent with previous cohort studies (41), we did not find that ACEIs (another RAAS inhibitor) were significantly associated with fracture risk or BMD. A recent meta-analysis also indicated that ACEIs use was not significantly associated with fracture risk, while ARBs use was associated with a lower risk of fracture (42). The mechanisms underlying the differences in the effects of the two RAAS inhibitors remain to be further investigated.

Previous researches on the relationships between CCBs and fracture risk and BMD were inconclusive. A Danish case-control study found a 6% reduction in fracture risk with CCBs use (43), while another study found an association between CCBs use and increased fracture risk (44). A large cohort study illustrated that there was no significant association between CCBs use and hip fracture risk (41). CCBs may affect bone metabolism, as they act by interfering with the transport of calcium through the cell membrane (45). Altering the activity of L-type calcium channels through drugs may modulate growth and differentiation of osteoblasts and stimulates the function of these cells (46). Some animal studies have suggested that CCBs may interfere with the production of steroid hormones and affect the process of bone repair and remodeling (47, 48), which may partly explain our results. However, some other studies pointed out that such agents may lead to reduced bone resorption and formation by directly or indirectly inhibiting the function and activity of osteoclasts (49, 50). Further mechanism studies were required to clarify the effects of CCBs on bone metabolism.

Thiazide diuretics, as one of the most common antihypertensive drugs, were generally considered to have a beneficial effect on BMD (51). Several RCTs indicated that thiazide diuretics could increase BMD in people with a high risk of osteoporosis compared to placebo (52, 53). Thiazide diuretics slowed bone loss by reducing urinary calcium excretion and stimulated osteoblast differentiation and bone mineral formation (54). Although many observational studies have assessed the potential associations between the use of thiazide diuretics and fracture risk, the findings remain to be inconsistent. An updated meta-analysis concluded that thiazide diuretics use was not a protective factor for fracture, given that thiazide diuretics were associated with a lower fracture risk only in case-control studies, rather than cohort studies (55). The metabolic alkalosis (56), hyponatremia (57), and upright hypotension (58) resulting from the use of thiazide diuretics may counteract their beneficial effects on calcium homeostasis and BMD, which may result in thiazide diuretics having little effect on the risk of osteoporotic fracture (59). Overall, thiazide diuretics could have a positive effect on BMD, although this effect is not always correlated with a decreased risk of fracture.

This study has several strengths. First, it used genetic variants mimicking antihypertensive drugs to examine the effect of drugs by drug target MR. This approach overcome limitations for observational studies, including reverse causality and potential confounders, while avoiding the time and resource constraints of RCTs. Second, the study utilized GWAS data sourced from the largest genetic studies to date, enhancing the statistical validity of the findings and conclusions. Third, genetic variants in the drug target genes associated with systolic blood pressure were first screened as proxies for antihypertensive drugs through strict selection procedures, followed by positive control analysis to ensure the validity of the genetic instruments. Fourth, various sensitivity analyses were conducted to validate reliability and consistency.

Our study is not without limitations. First, drug-target MR analysis evaluates long-term drug effects, which may have greater effect values than short-term drug effects in clinical trials. Therefore, this study may be more meaningful in providing direction for causal associations of drugs. Second, potential horizontal pleiotropy may affect the drug effects of MR analysis. We used instrumental variables near the genes encoding the drug targets, which minimized the possibility of pleiotropy. Also, there was no evidence of pleiotropy using several sensitivity analysis methods. Third, the association of genetically proxied ACEIs and CAD in positive control is not significant, which was similar to other MR studies (60, 61). This may indicate that the effect of ACEIs on CAD may be mediated through other pathways, not just the ACE target. Finally, our study data were confined to populations of European origin to ascertain genetic homogeneity. Hence, additional investigations and verification are warranted when the results are extrapolated to other ethnic groups with distinct genetic profiles.

## 5 Conclusion

The present study provided evidence for causal associations of antihypertensive drugs with fracture risk and BMD. These findings suggest that genetic proxies for ARBs and thiazide diuretics may have a protective effect on bone health, while genetic proxies for CCBs and PSDs may have a negative effect. This study supports the repurposing of antihypertensive drugs in the field of bone health and helps develop beneficial hypertension drug regimens for people with poor bone health in clinical practice.

## Data availability statement

The original contributions presented in the study are included in the article/**Supplementary Material**. Further inquiries can be directed to the corresponding authors.

## Author contributions

ZY and MZ participated in conceiving and designing the study. XH and TZ completed the statistical analysis and drafted the manuscript. PG, WG, and HZ provided valuable advice during the writing of the manuscript. All authors contributed to the article and approved the submitted version.

## References

[1] Hansson L Lindholm LH Niskanen L Lanke J Hedner T Niklason A. Effect of angiotensin-converting-enzyme inhibition compared with conventional therapy on cardiovascular morbidity and mortality in hypertension: The captopril prevention project (CAPPP) randomised trial. Lancet (1999) 353:611–6. doi: 10.1016/s0140-6736(98)05012-0 10030325

[2] Blackburn DF Wilson TW. Antihypertensive medications and blood sugar: Theories and implications. Can J Cardiol (2006) 22:229–33. doi: 10.1016/s0828-282x(06)70902-3 PMC2528930 16520854

[3] Butt DA Mamdani M Gomes T Lix L Lu H Tu K. Risk of osteoporotic fractures with angiotensin II receptor blockers versus angiotensin-converting enzyme inhibitors in hypertensive community-dwelling elderly. J Bone Miner Res (2014) 29:2483–8. doi: 10.1002/jbmr.2271 24806397

[4] Kwok T Leung J Barrett-Connor E Osteoporotic Fractures in Men (MrOS) Research Group. ARB users exhibit a lower fracture incidence than ACE inhibitor users among older hypertensive men. Age Ageing (2017) 46:57–64. doi: 10.1093/ageing/afw150 28181652 PMC5968636

[5] Langerhuizen DWG Verweij LPE van der Wouden JC Kerkhoffs GMMJ Janssen SJ. Antihypertensive drugs demonstrate varying levels of hip fracture risk: A systematic review and meta-analysis. Injury (2022) 53:1098–107. doi: 10.1016/j.injury.2021.09.036 34627629

[6] Klein D Nagel G Kleiner A Ulmer H Rehberger B Concin H. Blood pressure and falls in community-dwelling people aged 60 years and older in the VHM&PP cohort. BMC Geriatr (2013) 13:50. doi: 10.1186/1471-2318-13-50 23692779 PMC3663706

[7] Tinetti ME Han L Lee DSH McAvay GJ Peduzzi P Gross CP. Antihypertensive medications and serious fall injuries in a nationally representative sample of older adults. JAMA Intern Med (2014) 174:588–95. doi: 10.1001/jamainternmed.2013.14764 PMC4136657 24567036

[8] van der Baan FH Klungel OH Egberts ACG Leufkens HG Grobbee DE Roes KCB. Pharmacogenetics in randomized controlled trials: considerations for trial design. Pharmacogenomics (2011) 12:1485–92. doi: 10.2217/pgs.11.95 22008051

[9] Holmes MV Ala-Korpela M Smith GD. Mendelian randomization in cardiometabolic disease: challenges in evaluating causality. Nat Rev Cardiol (2017) 14:577–90. doi: 10.1038/nrcardio.2017.78 PMC5600813 28569269

[10] Walker VM Davey Smith G Davies NM Martin RM. Mendelian randomization: a novel approach for the prediction of adverse drug events and drug repurposing opportunities. Int J Epidemiol (2017) 46:2078–89. doi: 10.1093/ije/dyx207 PMC5837479 29040597

[11] Liu L Zeng P Xue F Yuan Z Zhou X. Multi-trait transcriptome-wide association studies with probabilistic mendelian randomization. Am J Hum Genet (2021) 108:240–56. doi: 10.1016/j.ajhg.2020.12.006 PMC7895847 33434493

[12] Ference BA Robinson JG Brook RD Catapano AL Chapman MJ Neff DR. Variation in PCSK9 and HMGCR and risk of cardiovascular disease and diabetes. N Engl J Med (2016) 375:2144–53. doi: 10.1056/NEJMoa1604304 27959767

[13] Williams B Mancia G Spiering W Agabiti Rosei E Azizi M Burnier M. 2018 ESC/ESH guidelines for the management of arterial hypertension. Eur Heart J (2018) 39:3021–104. doi: 10.1093/eurheartj/ehy339 30165516

[14] Skrivankova VW Richmond RC Woolf BAR Yarmolinsky J Davies NM Swanson SA. Strengthening the reporting of observational studies in epidemiology using mendelian randomization: The STROBE-MR statement. JAMA (2021) 326:1614–21. doi: 10.1001/jama.2021.18236 34698778

[15] Morris JA Kemp JP Youlten SE Laurent L Logan JG Chai RC. An atlas of genetic influences on osteoporosis in humans and mice. Nat Genet (2019) 51:258–66. doi: 10.1038/s41588-018-0302-x PMC6358485 30598549

[16] Medina-Gomez C Kemp JP Trajanoska K Luan J Chesi A Ahluwalia TS. Life-course genome-wide association study meta-analysis of total body BMD and assessment of age-specific effects. Am J Hum Genet (2018) 102:88–102. doi: 10.1016/j.ajhg.2017.12.005 29304378 PMC5777980

[17] Evangelou E Warren HR Mosen-Ansorena D Mifsud B Pazoki R Gao H. Genetic analysis of over 1 million people identifies 535 new loci associated with blood pressure traits. Nat Genet (2018) 50:1412–25. doi: 10.1038/s41588-018-0205-x PMC6284793 30224653

[18] van der Harst P Verweij N. Identification of 64 novel genetic loci provides an expanded view on the genetic architecture of coronary artery disease. Circ Res (2018) 122:433–43. doi: 10.1161/CIRCRESAHA.117.312086 PMC5805277 29212778

[19] Wishart DS Feunang YD Guo AC Lo EJ Marcu A Grant JR. DrugBank 5.0: a major update to the DrugBank database for 2018. Nucleic Acids Res (2018) 46:D1074–82. doi: 10.1093/nar/gkx1037 PMC5753335 29126136

[20] Huang W Xiao J Ji J Chen L. Association of lipid-lowering drugs with COVID-19 outcomes from a mendelian randomization study. Elife (2021) 10:e73873. doi: 10.7554/eLife.73873 34866576 PMC8709572

[21] Shim H Chasman DI Smith JD Mora S Ridker PM Nickerson DA. A multivariate genome-wide association analysis of 10 LDL subfractions, and their response to statin treatment, in 1868 caucasians. PloS One (2015) 10:e0120758. doi: 10.1371/journal.pone.0120758 25898129 PMC4405269

[22] Hemani G Tilling K Davey Smith G. Orienting the causal relationship between imprecisely measured traits using GWAS summary data. PloS Genet (2017) 13:e1007081. doi: 10.1371/journal.pgen.1007081 29149188 PMC5711033

[23] Xie W Zheng F Evangelou E Liu O Yang Z Chan Q. Blood pressure-lowering drugs and secondary prevention of cardiovascular disease: Systematic review and meta-analysis. J Hypertens (2018) 36:1256–65. doi: 10.1097/HJH.0000000000001720 29543625

[24] Wright JM Musini VM Gill R. First-line drugs for hypertension. Cochrane Database Syst Rev (2018) 2018:CD001841. doi: 10.1002/14651858.CD001841.pub3 PMC6513559 29667175

[25] Bowden J Davey Smith G Haycock PC Burgess S. Consistent estimation in mendelian randomization with some invalid instruments using a weighted median estimator. Genet Epidemiol (2016) 40:304–14. doi: 10.1002/gepi.21965 PMC4849733 27061298

[26] Slob EAW Burgess S. A comparison of robust mendelian randomization methods using summary data. Genet Epidemiol (2020) 44:313–29. doi: 10.1002/gepi.22295 PMC7317850 32249995

[27] Zhao Q Wang J Hemani G Bowden J Small DS. Statistical inference in two-sample summary-data mendelian randomization using robust adjusted profile score. Ann Stat (2020) 48:1742–69. doi: 10.1214/19-AOS1866

[28] Verbanck M Chen C-Y Neale B Do R. Detection of widespread horizontal pleiotropy in causal relationships inferred from mendelian randomization between complex traits and diseases. Nat Genet (2018) 50:693–8. doi: 10.1038/s41588-018-0099-7 PMC6083837 29686387

[29] Yavorska OO Burgess S. MendelianRandomization: an r package for performing mendelian randomization analyses using summarized data. Int J Epidemiol (2017) 46:1734–9. doi: 10.1093/ije/dyx034 PMC5510723 28398548

[30] Bowden J Davey Smith G Burgess S. Mendelian randomization with invalid instruments: effect estimation and bias detection through egger regression. Int J Epidemiol (2015) 44:512–25. doi: 10.1093/ije/dyv080 PMC4469799 26050253

[31] Staley JR Blackshaw J Kamat MA Ellis S Surendran P Sun BB. PhenoScanner: A database of human genotype-phenotype associations. Bioinformatics (2016) 32:3207–9. doi: 10.1093/bioinformatics/btw373 PMC5048068 27318201

[32] Kamat MA Blackshaw JA Young R Surendran P Burgess S Danesh J. PhenoScanner V2: An expanded tool for searching human genotype-phenotype associations. Bioinformatics (2019) 35:4851–3. doi: 10.1093/bioinformatics/btz469 PMC6853652 31233103

[33] Pouresmaeili F Kamalidehghan B Kamarehei M Goh YM. A comprehensive overview on osteoporosis and its risk factors. Ther Clin Risk Manag (2018) 14:2029–49. doi: 10.2147/TCRM.S138000 PMC6225907 30464484

[34] Carbone LD Vasan S Prentice RL Harshfield G Haring B Cauley JA. The renin-angiotensin aldosterone system and osteoporosis: findings from the women’s health initiative. Osteoporos Int (2019) 30:2039–56. doi: 10.1007/s00198-019-05041-3 31209511

[35] Ruths S Bakken MS Ranhoff AH Hunskaar S Engesæter LB Engeland A. Risk of hip fracture among older people using antihypertensive drugs: a nationwide cohort study. BMC Geriatr (2015) 15:153. doi: 10.1186/s12877-015-0154-5 26626043 PMC4667446

[36] Hatton R Stimpel M Chambers TJ. Angiotensin II is generated from angiotensin I by bone cells and stimulates osteoclastic bone resorption. vitro J Endocrinol (1997) 152:5–10. doi: 10.1677/joe.0.1520005 9014834

[37] Izu Y Mizoguchi F Kawamata A Hayata T Nakamoto T Nakashima K. Angiotensin II type 2 receptor blockade increases bone mass. J Biol Chem (2009) 284:4857–64. doi: 10.1074/jbc.M807610200 PMC2742875 19004830

[38] Shuai B Yang YP Shen L Zhu R Xu XJ Ma C. Local renin-angiotensin system is associated with bone mineral density of glucocorticoid-induced osteoporosis patients. Osteoporos Int (2015) 26:1063–71. doi: 10.1007/s00198-014-2992-y 25516362

[39] Shimizu H Nakagami H Osako MK Hanayama R Kunugiza Y Kizawa T. Angiotensin II accelerates osteoporosis by activating osteoclasts. FASEB J (2008) 22:2465–75. doi: 10.1096/fj.07-098954 18256306

[40] Garcia P Schwenzer S Slotta JE Scheuer C Tami AE Holstein JH. Inhibition of angiotensin-converting enzyme stimulates fracture healing and periosteal callus formation - role of a local renin-angiotensin system. Br J Pharmacol (2010) 159:1672–80. doi: 10.1111/j.1476-5381.2010.00651.x PMC2925490 20233225

[41] Bokrantz T Schiöler L Boström KB Kahan T Mellström D Ljungman C. Antihypertensive drug classes and the risk of hip fracture: results from the Swedish primary care cardiovascular database. J Hypertens (2020) 38:167–75. doi: 10.1097/HJH.0000000000002245 31568060

[42] Wu J Wang M Guo M Du X-Y Tan X-Z Teng F-Y. Angiotensin receptor blocker is associated with a lower fracture risk: An updated systematic review and meta-analysis. Int J Clin Pract (2022) 2022:7581110. doi: 10.1155/2022/7581110 35910069 PMC9303078

[43] Rejnmark L Vestergaard P Mosekilde L. Treatment with beta-blockers, ACE inhibitors, and calcium-channel blockers is associated with a reduced fracture risk: a nationwide case-control study. J Hypertens (2006) 24:581–9. doi: 10.1097/01.hjh.0000203845.26690.cb 16467662

[44] Takaoka S Yamaguchi T Tanaka K-I Morita M Yamamoto M Yamauchi M. Fracture risk is increased by the complication of hypertension and treatment with calcium channel blockers in postmenopausal women with type 2 diabetes. J Bone Miner Metab (2013) 31:102–7. doi: 10.1007/s00774-012-0389-6 23073638

[45] Moraes RB Corrêa L Luz JGC. Adverse effects of the amlodipine on bone healing of the mandibular fracture: An experimental study in rats. Oral Maxillofac Surg (2011) 15:93–101. doi: 10.1007/s10006-010-0237-6 20665062

[46] Nishiya Y Kosaka N Uchii M Sugimoto S. A potent 1,4-dihydropyridine l-type calcium channel blocker, benidipine, promotes osteoblast differentiation. Calcif Tissue Int (2002) 70:30–9. doi: 10.1007/s00223-001-1010-5 11907705

[47] Prakasam G Yeh JK Chen MM Castro-Magana M Liang CT Aloia JF. Effects of growth hormone and testosterone on cortical bone formation and bone density in aged orchiectomized rats. Bone (1999) 24:491–7. doi: 10.1016/s8756-3282(99)00018-6 10321909

[48] Almeida SA Teófilo JM Anselmo Franci JA Brentegani LG Lamano-Carvalho TL. Antireproductive effect of the calcium channel blocker amlodipine in male rats. Exp Toxicol Pathol (2000) 52:353–6. doi: 10.1016/S0940-2993(00)80062-7 10987190

[49] Ushijima K Liu Y Maekawa T Ishikawa E Motosugi Y Ando H. Protective effect of amlodipine against osteoporosis in stroke-prone spontaneously hypertensive rats. Eur J Pharmacol (2010) 635:227–30. doi: 10.1016/j.ejphar.2010.02.039 20193679

[50] Gradosova I Zivna H Palicka V Hubena S Svejkovska K Zivny P. Protective effect of amlodipine on rat bone tissue after orchidectomy. Pharmacology (2012) 89:37–43. doi: 10.1159/000335491 22302040

[51] Ghosh M Majumdar SR. Antihypertensive medications, bone mineral density, and fractures: a review of old cardiac drugs that provides new insights into osteoporosis. Endocrine (2014) 46:397–405. doi: 10.1007/s12020-014-0167-4 24504763

[52] LaCroix AZ Ott SM Ichikawa L Scholes D Barlow WE. Low-dose hydrochlorothiazide and preservation of bone mineral density in older adults. a randomized, double-blind, placebo-controlled trial. Ann Intern Med (2000) 133:516–26. doi: 10.7326/0003-4819-133-7-200010030-00010 11015164

[53] Reid IR Ames RW Orr-Walker BJ Clearwater JM Horne AM Evans MC. Hydrochlorothiazide reduces loss of cortical bone in normal postmenopausal women: A randomized controlled trial. Am J Med (2000) 109:362–70. doi: 10.1016/s0002-9343(00)00510-6 11020392

[54] Dvorak MM De Joussineau C Carter DH Pisitkun T Knepper MA Gamba G. Thiazide diuretics directly induce osteoblast differentiation and mineralized nodule formation by interacting with a sodium chloride co-transporter in bone. J Am Soc Nephrol (2007) 18:2509–16. doi: 10.1681/ASN.2007030348 PMC2216427 17656470

[55] Charkos TG Liu Y Jin L Yang S. Thiazide use and fracture risk: An updated Bayesian meta-analysis. Sci Rep (2019) 9:19754. doi: 10.1038/s41598-019-56108-4 31874989 PMC6930249

[56] Arnett TR Spowage M. Modulation of the resorptive activity of rat osteoclasts by small changes in extracellular pH near the physiological range. Bone (1996) 18:277–9. doi: 10.1016/8756-3282(95)00486-6 8703584

[57] Hovis JG Meyer T Teasdale RM Albrecht BN Yorek MA Lowe WL. Intracellular calcium regulates insulin-like growth factor-I messenger ribonucleic acid levels. Endocrinology (1993) 132:1931–8. doi: 10.1210/endo.132.5.8477645 8477645

[58] Hartog LC Schrijnders D Landman GWD Groenier K Kleefstra N Bilo HJG. Is orthostatic hypotension related to falling? a meta-analysis of individual patient data of prospective observational studies. Age Ageing (2017) 46:568–75. doi: 10.1093/ageing/afx024 PMC6402310 28338807

[59] Wang J Su K Sang W Li L Ma S. Thiazide diuretics and the incidence of osteoporotic fracture: A systematic review and meta-analysis of cohort studies. Front Pharmacol (2019) 10:1364. doi: 10.3389/fphar.2019.01364 31824314 PMC6881387

[60] Gill D Georgakis MK Koskeridis F Jiang L Feng Q Wei W-Q. Use of genetic variants related to antihypertensive drugs to inform on efficacy and side effects. Circulation (2019) 140:270–9. doi: 10.1161/CIRCULATIONAHA.118.038814 PMC6687408 31234639

[61] Yarmolinsky J Díez-Obrero V Richardson TG Pigeyre M Sjaarda J Paré G. Genetically proxied therapeutic inhibition of antihypertensive drug targets and risk of common cancers: A mendelian randomization analysis. PloS Med (2022) 19:e1003897. doi: 10.1371/journal.pmed.1003897 35113855 PMC8812899

