# Supplementary material for: Association of antihypertensive drugs with fracture and bone mineral density: A comprehensive drug-target Mendelian randomization study
Source: Front Endocrinol (Lausanne). 2023 Mar 28;14:1164387. doi: 10.3389/fendo.2023.1164387 (PMC10086430; doi:10.3389/fendo.2023.1164387)
Supplement: Supplementary file 1 [file Image_1.pdf]

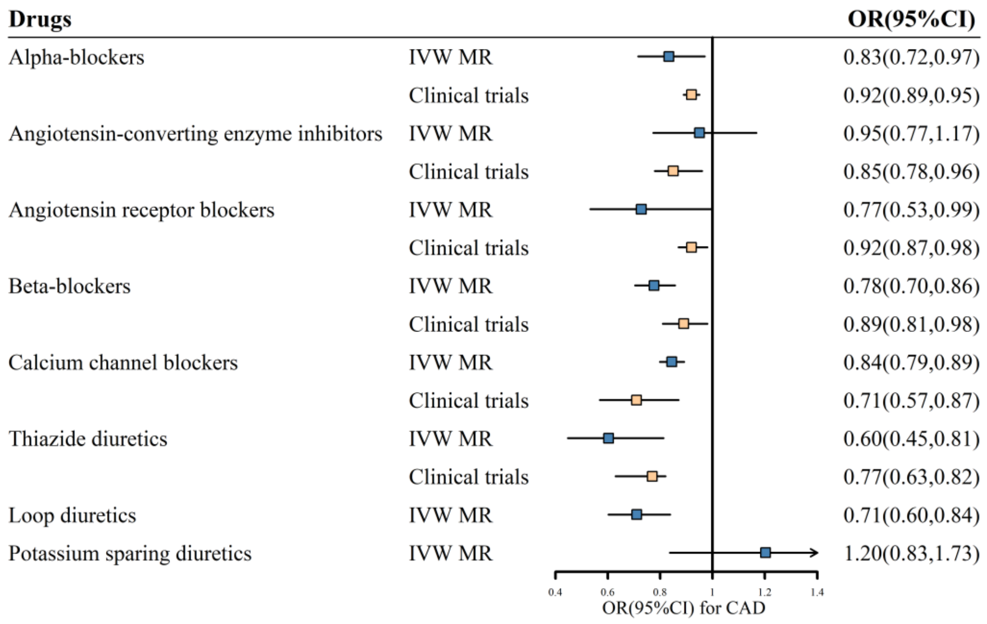

Supplementary Figure 1. MR estimates for the effect of antihypertensive drugs on risk of CAD CI, confidence interval; OR, odds ratio; IVW, inverse-variance weighted; MR, Mendelian Randomization; CAD, coronary artery disease.

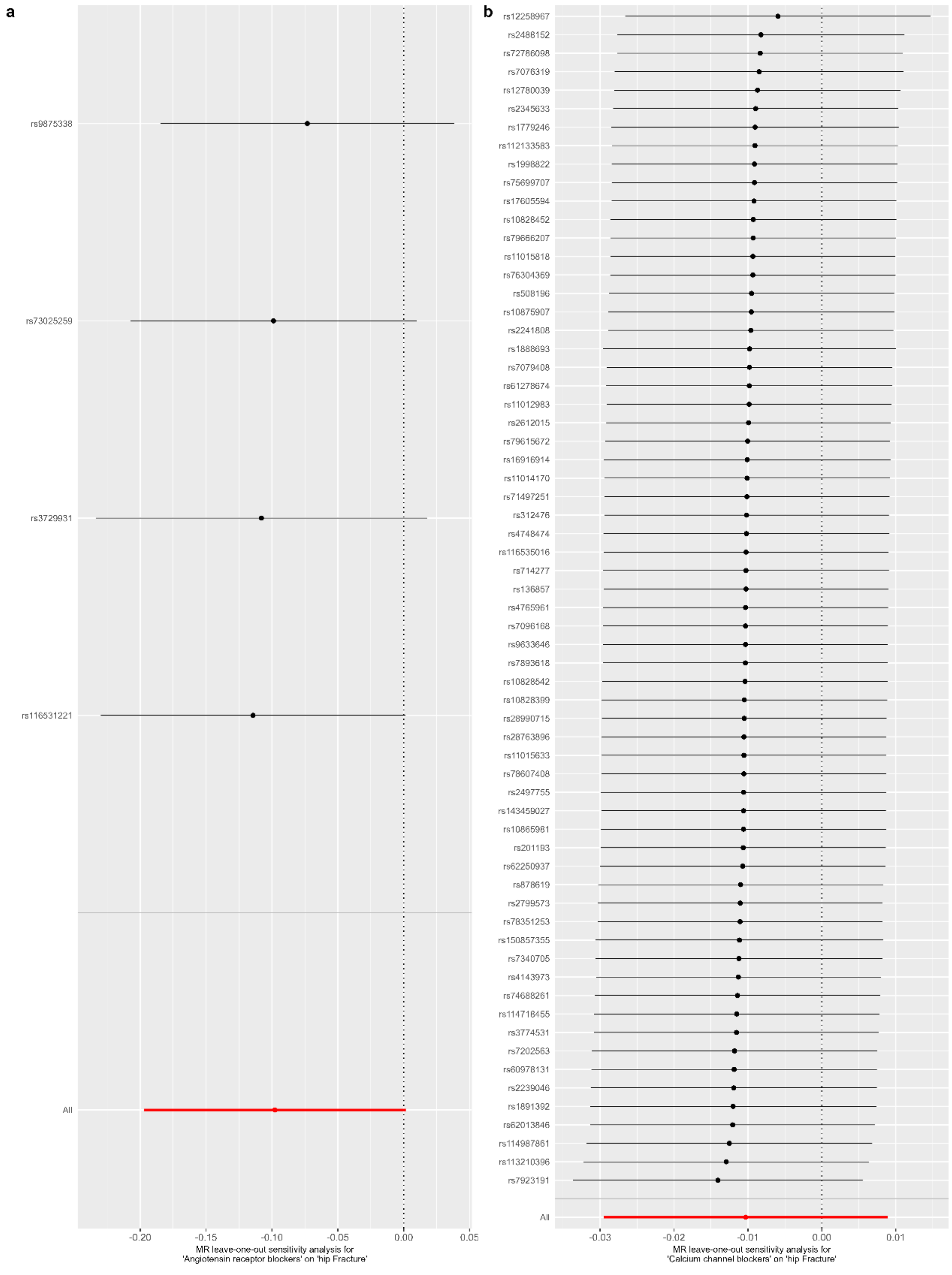

Supplementary Figure 2. Leave-one-out plot to assess if a single variant is driving the association between ARBs, CCBs and Fracture(a)Leave-one-out plot to assess if a single variant is driving the association between ARBs and Fracture. (b) Leave-one-out plot to assess if a single variant is driving the association between CCBs and Fracture. ARBs, angiotensin receptor blockers; CCBs, calcium channel blockers.

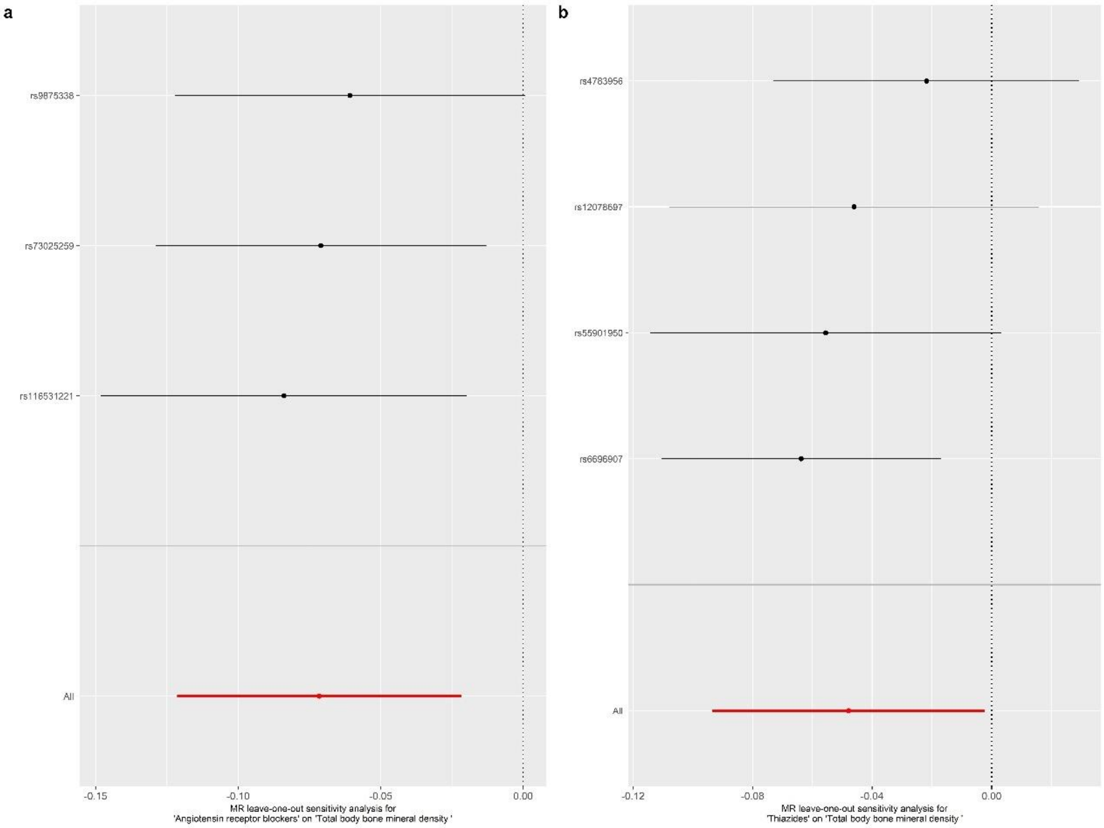

Supplementary Figure 3. Leave-one-out plot to assess if a single variant is driving the association between ARBs, thiazides diuretics and TB-BMD. (a) Leave-one-out plot to assess if a single variant is driving the association between ARBs and TB-BMD. (b) Leave-one-out plot to assess if a single variant is driving the association between thiazides diuretics and TB-BMD. ARBs, angiotensin receptor blockers; TB-BMD, total body bone mineral density.

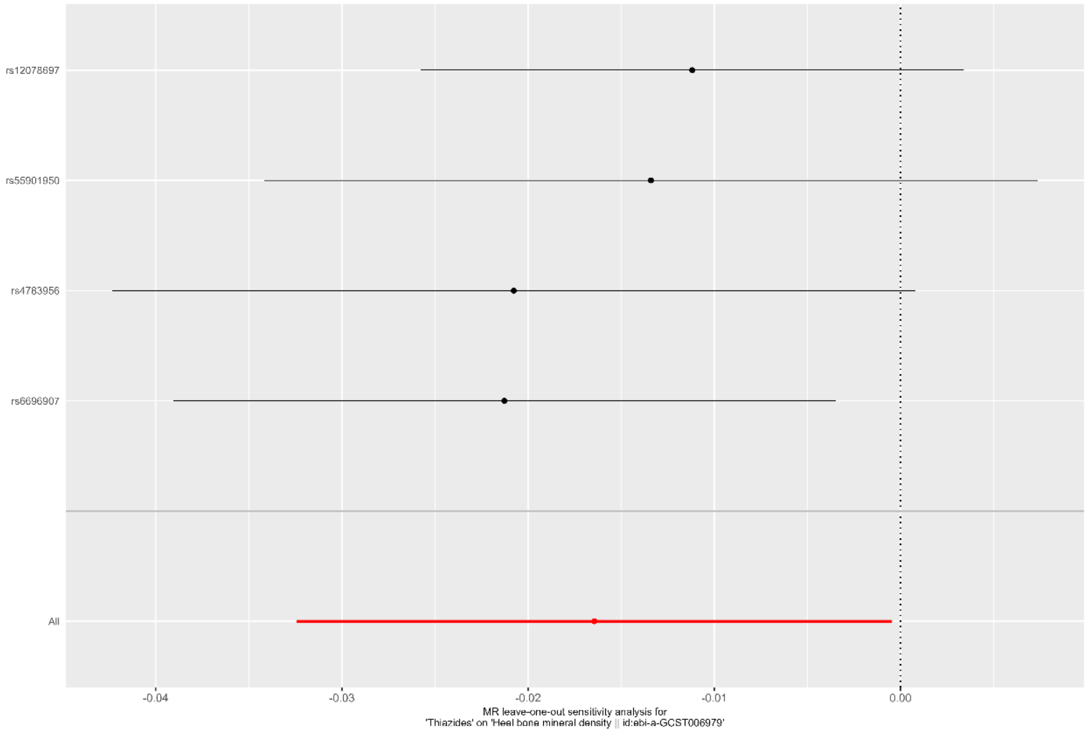

Supplementary Figure 4. Leave-one-out plot to assess if a single variant is driving the association between thiazides diuretics and eBMD. eBMD, estimated heel bone mineral density.
