# Supplementary material for: Association of antihypertensive drugs with fracture and bone mineral density: A comprehensive drug-target Mendelian randomization study
Source: Front Endocrinol (Lausanne). 2023 Mar 28;14:1164387. doi: 10.3389/fendo.2023.1164387 (PMC10086430; doi:10.3389/fendo.2023.1164387)
Supplement: Supplementary file 2 [file Table_1.pdf]

STROBE-MR checklist of recommended items to address in reports of Mendelian randomization studies

Item No.	Section		Checklist item	Page No.	Relevant text from manuscript
1	**TITLE and ABSTRACT**		Indicate Mendelian randomization (MR) as the study's design in the title and/or the abstract if that is a main purpose of the study	1-2	Complete
	**INTRODUCTION**				
2	**Background**		Explain the scientific background and rationale for the reported study. What is the exposure? Is a potential causal relationship between exposure and outcome plausible? Justify why MR is a helpful method to address the study question	2	Complete - Concept of Mendelian randomization and specific request for drug target Mendelian randomization were explained in the 2-3 paragraph of the introduction.
3	**Objectives**		State specific objectives clearly, including pre-specified causal hypotheses (if any). State that MR is a method that, under specific assumptions, intends to estimate causal effects	2	Complete - The causal question has been stated in the paragraph 4 of the introduction.
	**METHODS**				
4	**Study design and data sources**		Present key elements of the study design early in the article. Consider including a table listing sources of data for all phases of the study. For each data source contributing to the analysis, describe the following:		
		a)	Setting: Describe the study design and the underlying population, if possible. Describe the setting, locations, and relevant dates, including periods of recruitment, exposure, follow-up, and data collection, when available.	3	Complete - All necessary information about the study design and the underlying population been used in this study have been described in the study design and Data source section.
		b)	Participants: Give the eligibility criteria, and the sources and methods of selection of participants. Report the sample size, and whether any power or sample size calculations were carried out prior to the main analysis	3	Complete - All necessary information about the GWAS studies been used in this study have been described in the method section and Table 1.
		c)	Describe measurement, quality control and selection of genetic variants	3-4	Complete - The genetic predictor selection process has been described in the Methods section "Genetic instrument selection for antihypertensive drugs" and in Figure 1.
		d)	For each exposure, outcome, and other relevant variables, describe methods of assessment and diagnostic criteria for diseases	3	Complete - The information about the diagnostic criteria for diseases in this study have been described in the method section.
		e)	Provide details of ethics committee approval and participant informed consent, if relevant	3	Complete - Ethics approval and informed consent info in the "Ethics statement" section.
5	**Assumptions**		Explicitly state the three core IV assumptions for the main analysis (relevance, independence and exclusion restriction) as well assumptions for any additional or sensitivity analysis		Complete - The Mendelian randomization assumptions have been described in Figure 1A.

6	**Statistical methods: main analysis**		Describe statistical methods and statistics used		
		a)	Describe how quantitative variables were handled in the analyses (i.e., scale, units, model)	3	Complete - Described in methods and Figure 1.
		b)	Describe how genetic variants were handled in the analyses and, if applicable, how their weights were selected	3	Complete - Described in the section "Genetic instrument selection for antihypertensive drugs" within Methods.
		c)	Describe the MR estimator (e.g. two-stage least squares, Wald ratio) and related statistics. Detail the included covariates and, in case of two-sample MR, whether the same covariate set was used for adjustment in the two samples	4	Complete - Described in the section "Statistical analyses" within Methods.
		d)	Explain how missing data were addressed	4	Complete - Described in the section "Statistical analyses" within Methods.
		e)	If applicable, indicate how multiple testing was addressed	4	Complete - Described in the section "Statistical analyses" within Methods.
7	**Assessment of assumptions**		Describe any methods or prior knowledge used to assess the assumptions or justify their validity	4	Complete - Described in the section "Statistical analyses" within Methods.
8	**Sensitivity analyses and additional analyses**		Describe any sensitivity analyses or additional analyses performed (e.g. comparison of effect estimates from different approaches, independent replication, bias analytic techniques, validation of instruments, simulations)	4-5	Complete - The Mendelian randomization sensitivity analyses have been listed in "Sensitivity analysis" section of the Method.
9	**Software and pre-registration**				
		a)	Name statistical software and package(s), including version and settings used	4	Complete - All statistical software and settings used are described in the "Statistical analysis" section.
		b)	State whether the study protocol and details were pre-registered (as well as when and where)	3	Complete - The analysis plan was described in the "Study design" section of the Methods and Figure 1.
	**RESULTS**				
10	**Descriptive data**				
		a)	Report the numbers of individuals at each stage of included studies and reasons for exclusion. Consider use of a flow diagram	3	Complete - Information is given in the "Data source" section of the Methods and Table 1.
		b)	Report summary statistics for phenotypic exposure(s), outcome(s), and other relevant variables (e.g. means, SDs, proportions)		Complete - We listed the detailed information of the summary statistics for our instruments in Supplementary Table 2.
		c)	If the data sources include meta-analyses of previous studies, provide the assessments of heterogeneity across these studies		NA.

		d)	For two-sample MR:<br>i. Provide justification of the similarity of the genetic variant-exposure associations between the exposure and outcome samples<br>ii. Provide information on the number of individuals who overlap between the exposure and outcome studies	3	Complete - We provide this information in the "Data source" section of the Methods.
11	**Main results**				
		a)	Report the associations between genetic variant and exposure, and between genetic variant and outcome, preferably on an interpretable scale		Complete - Genetic exposure associations have been reported in Supplementary Table 2.
		b)	Report MR estimates of the relationship between exposure and outcome, and the measures of uncertainty from the MR analysis, on an interpretable scale, such as odds ratio or relative risk per SD difference	5-6	Complete - The causal effect estimates between exposures and outcomes were listed in Figure 2-4 and Supplementary Table 4-6.
		c)	If relevant, consider translating estimates of relative risk into absolute risk for a meaningful time period	5-6	Complete - Our results were presented in terms of odds ratio and confidence intervals throughout the results section for binary outcomes and as beta coefficient for quantitative outcomes.
		d)	Consider plots to visualize results (e.g. forest plot, scatterplot of associations between genetic variants and outcome versus between genetic variants and exposure)		Complete – See Figure 2-3 and Supplementary Figure 1.
12	**Assessment of assumptions**				
		a)	Report the assessment of the validity of the assumptions	5 -6	Complete –We assess the validity using IVW, weighted median method, MR-Robust, MR-RAPS, MR-Lasso, and MR-PRESSO. Results were presented in Results.
		b)	Report any additional statistics (e.g., assessments of heterogeneity across genetic variants, such as I^2, Q statistic or E-value)	5-6	Complete –We report the use of Egger intercept, Cochran's Q in the Results and Supplementary Table 3.
13	**Sensitivity analyses and additional analyses**				
		a)	Report any sensitivity analyses to assess the robustness of the main results to violations of the assumptions	5-6	Complete – We used IVW, weighted median method, MR-Robust, MR-RAPS, MR-Lasso, and MR-PRESSO as sensitivity analyses. See Supplementary Table 4-6.
		b)	Report results from other sensitivity analyses or additional analyses	5-6	Complete – We conducted leave-one-out analysis and excluded identified pleiotropic SNPs. See Supplementary Table 7-8 and Supplementary Figure 2-4.
		c)	Report any assessment of direction of causal relationship (e.g., bidirectional MR)		NA.

		d)	When relevant, report and compare with estimates from non-MR analyses		NA
		e)	Consider additional plots to visualize results (e.g., leave-one-out analyses)		Complete – See Supplementary Figure 2-4.
	**DISCUSSION**				
14	**Key results**		Summarize key results with reference to study objectives	6	Complete – Discussion paragraph 1.
15	**Limitations**		Discuss limitations of the study, taking into account the validity of the IV assumptions, other sources of potential bias, and imprecision. Discuss both direction and magnitude of any potential bias and any efforts to address them	8	Complete – Discussion paragraph 6.
16	**Interpretation**				
		a)	Meaning: Give a cautious overall interpretation of results in the context of their limitations and in comparison with other studies	6-7	Complete – Discussion paragraph 2-4.
		b)	Mechanism: Discuss underlying biological mechanisms that could drive a potential causal relationship between the investigated exposure and the outcome, and whether the gene-environment equivalence assumption is reasonable. Use causal language carefully, clarifying that IV estimates may provide causal effects only under certain assumptions	6-7	Complete – Discussion paragraph 2-4.
		c)	Clinical relevance: Discuss whether the results have clinical or public policy relevance, and to what extent they inform effect sizes of possible interventions	6-7	Complete – Discussion paragraph 2-4.
17	**Generalizability**		Discuss the generalizability of the study results (a) to other populations, (b) across other exposure periods/timings, and (c) across other levels of exposure	8	Complete – Discussion paragraph 6.
	**OTHER INFORMATION**				
18	**Funding**		Describe sources of funding and the role of funders in the present study and, if applicable, sources of funding for the databases and original study or studies on which the present study is based	8	Complete – We have reported all sources of funding in the "Funding" section.
19	**Data and data sharing**		Provide the data used to perform all analyses or report where and how the data can be accessed, and reference these sources in the article. Provide the statistical code needed to reproduce the results in the article, or report whether the code is publicly accessible and if so, where	8	Complete – We have provided the link/approach to access genetic data used in this study in the "Data sharing statement" section.
20	**Conflicts of Interest**		All authors should declare all potential conflicts of interest	9	Complete –The authors declare that the research was conducted in the absence of any commercial or financial relationships that could be construed as a potential conflict of interest.

This checklist is copyrighted by the Equator Network under the Creative Commons Attribution 3.0 Unported (CC BY 3.0) license.
