# Supplementary material for: Association of antihypertensive drugs with fracture and bone mineral density: A comprehensive drug-target Mendelian randomization study
Source: Front Endocrinol (Lausanne). 2023 Mar 28;14:1164387. doi: 10.3389/fendo.2023.1164387 (PMC10086430; doi:10.3389/fendo.2023.1164387)
Supplement: Supplementary file 3 [file Table_2.pdf]

Supplementary Table 1. Classes of antihypertensive drugs and target genes

Class	Target gene	Examples
Targeting renin–angiotensin system		
ACEIs	ACE	Captopril, lisinopril, ramipril
ARBs	AGTR1, PPAG	Candesartan, losartan, valsartan, telmisartan
Adrenoceptor antagonists		
BBs	ADRB1, ADRB2	Atenolol, metoprolol, propranolol
alpha-blockers	ADRA1A, ADRA1D, ADRA1B, ADRA2A, ADRA2B	Doxazosin, phentolamine, phenoxybenzamine
CCBs	CACNA1B, CACNA1C, CACNA1D, CACNA1F, CACNA1S, CACNB1, CACNB2, CACNB3, CACNB4, CACNA1A, CACNA1H, CACNA1G, CACNA1I, CACNG1, CACNA2D1, CACNA2D2	Amlodipine, nifedipine, nimodipine, benidipine, lercanidipine, isradipine
Diuretics		
thiazide diuretics	SLC12A3, KCNMA1, ATP1A1	Bendroflumethiazide, hydrochlorothiazide
loop diuretics	SLC12A1, SLC12A2, SLC12A4, SLC12A5	Furosemide, bumetanide
PSDs	SCNN1A, SCNN1B, SCNN1G, SCNN1D, NR3C2	Amiloride, spironolactone

ACEIs, angiotensin-converting enzyme inhibitors; ARBs, angiotensin receptor blockers; BBs, beta-blockers; CCBs, calcium channel blockers; PSDs, potassium sparing diuretics.

Supplementary Table 2. Genetic variants selected as instruments for different classes of antihypertensive drugs

Drugs	SNP	Chr	Pos	EA	EAF	Effect	SE	*P*	R^{2*}	F^{*}
ACEIs	rs28656895	17	61456256	T	0.23	-0.19	0.04	5.54E-08	4.07E-05	29.47
ACEIs	rs9899012	17	61545486	A	0.06	-0.30	0.07	8.48E-06	3.08E-05	19.85
ACEIs	rs8077276	17	61547562	A	0.62	-0.29	0.03	5.15E-21	1.22E-04	88.20
ACEIs	rs8066276	17	61589265	C	0.59	0.13	0.03	3.65E-05	2.37E-05	16.99
ACEIs	rs79480822	17	61654225	T	0.05	0.32	0.08	1.65E-05	2.99E-05	18.56
ARBs	rs9875338	3	12296469	A	0.40	-0.13	0.03	1.43E-05	2.57E-05	18.85
ARBs	rs73025259	3	12414290	T	0.04	0.30	0.08	9.77E-05	2.17E-05	15.20
ARBs	rs116531221	3	12554731	G	0.01	-0.65	0.14	2.93E-06	3.54E-05	21.85
ARBs	rs3729931	3	12626516	A	0.36	-0.18	0.03	6.29E-09	4.64E-05	33.71
BBs	rs75228369	10	115684914	T	0.05	0.29	0.07	9.18E-05	2.30E-05	15.30
BBs	rs7894582	10	115704458	A	0.05	-0.30	0.07	3.57E-05	2.36E-05	17.08
BBs	rs11196553	10	115710997	T	0.05	0.63	0.07	2.89E-17	9.91E-05	71.38
BBs	rs78793615	10	115718544	T	0.02	-0.54	0.11	9.46E-07	3.57E-05	24.03
BBs	rs460718	10	115721364	G	0.67	0.28	0.03	1.36E-17	1.00E-04	72.78
BBs	rs10787510	10	115747742	G	0.52	0.16	0.03	2.06E-07	3.84E-05	26.95
BBs	rs678669	10	115764032	T	0.61	-0.14	0.03	1.02E-05	2.72E-05	19.48
BBs	rs11196597	10	115788094	A	0.13	0.29	0.05	4.23E-10	5.55E-05	38.94
BBs	rs17875473	10	115800294	T	0.09	0.33	0.06	2.66E-09	5.00E-05	35.37
BBs	rs68122733	10	115831533	G	0.17	-0.33	0.04	1.69E-16	9.47E-05	67.80
BBs	rs143854972	10	115843445	A	0.06	0.43	0.07	8.89E-11	6.00E-05	42.05
BBs	rs11196627	10	115845762	C	0.39	0.16	0.03	9.39E-07	3.46E-05	24.07
BBs	rs12219192	10	116000453	T	0.16	0.18	0.05	4.73E-05	2.63E-05	16.52
BBs	rs1862341	5	148030150	C	0.24	-0.17	0.04	2.12E-06	3.10E-05	22.44
BBs	rs888956	5	148120217	C	0.23	0.15	0.04	5.98E-05	2.31E-05	16.09
BBs	rs10491337	5	148331046	T	0.05	-0.43	0.07	5.27E-09	4.76E-05	34.05
BBs	rs78909293	5	148335250	C	0.04	-0.38	0.08	5.15E-07	3.58E-05	25.24
BBs	rs17640858	5	148346260	A	0.37	0.20	0.03	3.30E-10	5.66E-05	39.36
BBs	rs994446	5	148348395	A	0.21	-0.35	0.04	1.87E-20	1.18E-04	85.88
BBs	rs77187693	5	148396965	G	0.03	-0.45	0.10	6.34E-06	2.97E-05	20.40
alpha-blockers	rs7826601	8	26410379	C	0.71	0.14	0.03	1.36E-05	2.57E-05	18.90
alpha-blockers	rs45471201	8	26515138	T	0.10	0.26	0.05	4.14E-07	3.61E-05	25.65
alpha-blockers	rs7835853	8	26662246	T	0.21	0.19	0.04	6.09E-07	3.40E-05	24.87
alpha-blockers	rs17296809	8	26706343	C	0.06	0.33	0.07	4.43E-07	3.50E-05	25.49
alpha-blockers	rs2046187	8	26812434	A	0.41	-0.14	0.03	3.28E-06	2.98E-05	21.65
alpha-blockers	rs2546963	5	159522488	T	0.58	0.12	0.03	5.41E-05	2.26E-05	16.26
alpha-blockers	rs116286399	5	159576612	A	0.06	-0.31	0.07	2.25E-06	3.28E-05	22.40
alpha-blockers	rs4815660	20	4095716	A	0.66	-0.17	0.03	3.48E-08	4.09E-05	30.37
alpha-blockers	rs1764975	20	4101290	A	0.80	0.28	0.04	1.08E-13	7.73E-05	55.32
alpha-blockers	rs6139317	20	4104510	A	0.36	0.14	0.03	1.50E-05	2.66E-05	18.75
alpha-blockers	rs297738	20	4413512	A	0.28	-0.14	0.03	3.06E-05	2.38E-05	17.38
alpha-blockers	rs11551400	2	96914636	C	0.19	-0.18	0.04	3.81E-06	2.95E-05	21.36
CCBs	rs4143973	19	13266839	T	0.51	-0.13	0.03	1.85E-05	2.49E-05	18.30
CCBs	rs143459027	12	2212867	T	0.03	0.44	0.10	4.36E-06	3.21E-05	21.11
CCBs	rs2239046	12	2434419	A	0.68	0.21	0.03	9.58E-11	5.66E-05	41.81
CCBs	rs714277	12	2514270	T	0.28	0.20	0.03	2.38E-09	4.81E-05	35.57

CCBs	rs4765961	12	2668472	C	0.17	-0.21	0.04	3.74E-07	3.55E-05	25.80
CCBs	rs28990715	12	2973547	T	0.02	0.44	0.11	7.03E-05	2.25E-05	15.80
CCBs	rs114718455	3	53464055	G	0.03	0.51	0.09	1.72E-08	4.96E-05	31.78
CCBs	rs312476	3	53514416	T	0.25	0.14	0.04	6.68E-05	2.23E-05	15.91
CCBs	rs114987861	3	53605712	A	0.03	0.53	0.10	3.36E-08	4.48E-05	30.48
CCBs	rs113210396	3	53612327	T	0.05	-0.43	0.08	1.76E-08	4.78E-05	31.74
CCBs	rs116535016	3	53645516	A	0.02	0.50	0.11	3.01E-06	3.14E-05	21.80
CCBs	rs7340705	3	53734443	C	0.33	0.24	0.03	4.87E-14	7.79E-05	56.72
CCBs	rs3774531	3	53737575	T	0.49	0.13	0.03	1.20E-05	2.60E-05	19.12
CCBs	rs2612015	3	53742331	T	0.63	0.13	0.03	8.03E-05	2.19E-05	15.55
CCBs	rs2241808	3	53856653	T	0.54	0.15	0.03	7.68E-07	3.38E-05	24.44
CCBs	rs62250937	3	53870318	C	0.09	-0.32	0.06	7.28E-09	5.10E-05	33.43
CCBs	rs60978131	3	53982904	T	0.02	0.58	0.14	5.71E-05	2.40E-05	16.19
CCBs	rs10865981	3	54003534	C	0.09	-0.26	0.05	1.45E-06	3.25E-05	23.24
CCBs	rs878619	17	48633271	G	0.38	-0.14	0.03	4.29E-06	2.88E-05	21.11
CCBs	rs62013846	16	1063999	C	0.04	-0.36	0.08	9.45E-06	3.02E-05	19.64
CCBs	rs7202563	16	1389153	A	0.91	-0.29	0.05	8.30E-08	4.15E-05	28.70
CCBs	rs136857	22	40064558	C	0.72	0.14	0.03	8.35E-05	2.19E-05	15.47
CCBs	rs28763896	3	50290016	A	0.05	-0.28	0.07	6.56E-05	2.32E-05	15.92
CCBs	rs201193	3	50643305	T	0.86	-0.22	0.04	5.83E-07	3.41E-05	24.95
CCBs	rs508196	10	18263873	T	0.43	-0.16	0.03	1.15E-07	3.93E-05	28.11
CCBs	rs2497755	10	18282667	T	0.89	-0.30	0.05	7.72E-10	5.16E-05	37.77
CCBs	rs7893618	10	18299520	G	0.57	-0.14	0.03	1.74E-05	2.98E-05	18.46
CCBs	rs1891392	10	18336421	C	0.71	-0.23	0.03	9.02E-12	6.35E-05	46.44
CCBs	rs2488152	10	18355456	G	0.10	0.38	0.05	2.54E-14	7.96E-05	57.97
CCBs	rs76304369	10	18359213	T	0.01	0.73	0.14	1.19E-07	3.92E-05	28.05
CCBs	rs75699707	10	18359294	A	0.03	-0.58	0.10	2.10E-08	4.90E-05	31.41
CCBs	rs78351253	10	18361384	G	0.04	-0.35	0.08	1.21E-05	2.71E-05	19.15
CCBs	rs7079408	10	18377343	G	0.31	-0.18	0.03	7.58E-08	3.98E-05	28.88
CCBs	rs78607408	10	18386117	C	0.05	-0.33	0.08	1.03E-05	2.79E-05	19.43
CCBs	rs74688261	10	18412294	G	0.07	0.27	0.06	3.11E-06	2.97E-05	21.75
CCBs	rs1888693	10	18440444	A	0.34	0.39	0.03	4.69E-34	2.02E-04	148.12
CCBs	rs16916914	10	18457722	C	0.04	0.56	0.08	2.72E-12	6.69E-05	48.90
CCBs	rs7076319	10	18459450	G	0.27	0.32	0.03	5.07E-21	1.21E-04	88.61
CCBs	rs17605594	10	18471064	T	0.06	-0.29	0.07	9.38E-05	2.58E-05	15.26
CCBs	rs61278674	10	18481737	G	0.09	0.33	0.05	1.03E-09	5.52E-05	37.30
CCBs	rs11012983	10	18490601	T	0.11	0.22	0.05	6.80E-06	2.81E-05	20.21
CCBs	rs1779246	10	18506911	A	0.80	-0.30	0.04	3.47E-15	8.57E-05	61.81
CCBs	rs10828399	10	18553968	A	0.52	-0.19	0.03	1.10E-10	5.67E-05	41.56
CCBs	rs9633646	10	18564922	A	0.59	-0.13	0.03	1.46E-05	2.57E-05	18.79
CCBs	rs79666207	10	18583840	C	0.02	-0.63	0.12	2.61E-07	3.99E-05	26.52
CCBs	rs10828452	10	18592450	T	0.21	-0.30	0.04	4.20E-15	9.07E-05	61.63
CCBs	rs2799573	10	18601928	C	0.28	0.14	0.03	5.44E-05	2.25E-05	16.26
CCBs	rs10828542	10	18627285	G	0.39	-0.18	0.03	5.18E-09	4.71E-05	34.13
CCBs	rs12780039	10	18678987	C	0.12	0.29	0.05	1.26E-09	5.17E-05	36.82
CCBs	rs7096168	10	18693174	A	0.20	0.18	0.04	2.20E-06	3.08E-05	22.41
CCBs	rs112133583	10	18695681	T	0.03	-0.55	0.10	1.18E-08	5.20E-05	32.49
CCBs	rs79615672	10	18700784	T	0.02	-0.48	0.11	5.83E-06	3.18E-05	20.53

CCBs	rs11014170	10	18710991	A	0.02	-0.67	0.12	5.61E-09	5.25E-05	33.95
CCBs	rs7923191	10	18727901	G	0.21	0.37	0.04	1.09E-22	1.35E-04	96.31
CCBs	rs12258967	10	18727959	G	0.30	-0.63	0.03	1.08E-78	4.95E-04	352.48
CCBs	rs72786098	10	18729855	A	0.03	-0.50	0.09	1.18E-08	4.61E-05	32.49
CCBs	rs1998822	10	18755664	G	0.28	0.20	0.03	1.15E-08	4.61E-05	32.59
CCBs	rs71497251	10	18781265	A	0.19	0.20	0.04	2.89E-07	3.77E-05	26.33
CCBs	rs4748474	10	18790727	A	0.52	0.19	0.03	1.61E-10	5.67E-05	40.98
CCBs	rs11015633	10	18979469	A	0.58	0.14	0.03	3.34E-06	2.99E-05	21.64
CCBs	rs11015818	10	19000055	T	0.61	0.13	0.03	3.86E-05	2.53E-05	16.95
CCBs	rs150857355	12	49209340	C	0.02	0.94	0.11	5.20E-17	1.08E-04	70.28
CCBs	rs10875907	12	49389410	A	0.02	-0.72	0.12	4.28E-09	5.55E-05	34.48
CCBs	rs2345633	2	152946604	C	0.40	-0.16	0.03	4.77E-07	3.48E-05	25.29
thiazide diuretics	rs4783956	16	56766831	T	0.29	-0.18	0.03	8.98E-08	3.85E-05	28.56
thiazide diuretics	rs55901950	16	56920146	A	0.08	-0.24	0.06	1.04E-05	2.69E-05	19.40
thiazide diuretics	rs6696907	1	116867355	G	0.73	-0.15	0.03	1.24E-05	2.72E-05	19.10
thiazide diuretics	rs12078697	1	117015118	C	0.21	-0.15	0.04	8.06E-05	2.15E-05	15.53
loop diuretics	rs2015637	15	48716853	C	0.10	-0.41	0.05	1.16E-15	8.80E-05	64.16
loop diuretics	rs13356360	5	127247719	C	0.04	0.35	0.09	5.96E-05	2.66E-05	16.10
loop diuretics	rs6860245	5	127367998	C	0.24	-0.21	0.04	2.10E-09	4.89E-05	35.80
loop diuretics	rs141600739	5	127565218	C	0.02	-0.41	0.10	6.33E-05	2.29E-05	16.01
loop diuretics	rs17676242	5	127589648	A	0.09	0.42	0.05	6.62E-15	8.58E-05	60.80
loop diuretics	rs3805639	5	127692822	G	0.11	0.20	0.05	2.40E-05	2.45E-05	17.85
loop diuretics	rs6130942	20	44464926	T	0.55	-0.15	0.03	6.47E-07	3.41E-05	24.77
loop diuretics	rs6074044	20	44782142	T	0.13	0.18	0.05	4.42E-05	2.32E-05	16.70
loop diuretics	rs2425773	20	44831696	T	0.72	0.13	0.03	9.05E-05	2.17E-05	15.29
PSDs	rs112615655	1	1187448	A	0.03	-0.39	0.10	5.75E-05	2.44E-05	16.16
PSDs	rs307359	1	1280014	G	0.93	0.38	0.06	5.17E-09	5.34E-05	34.14

*The R^2 is proportion of variance in phenotype explained by a given SNP. The R^2 and F statistic was calculated using the formula in previous study (21).

SNP, single nucleotide polymorphism; Chr, chromosome; Pos, Position; EA, effect allele; OA, other allele; EAF, effect allele frequency; SE, standard error; ACEIs, angiotensin-converting enzyme inhibitors; ARBs, angiotensin receptor blockers; BBs, beta-blockers; CCBs, calcium channel blockers; BBs, beta-blockers; PSDs, potassium sparing diuretics.

Supplementary Table 3. Heterogeneity and pleiotropy tests for drug target MR analyses

Exposure	Outcome	Method	Heterogeneity analysis			Pleiotropy analysis		
			Q	Degree of freedom	*P*	Egger intercept	SE	*P*
alpha-blockers	Fracture	MR Egger	10.83	10	0.371	0.030	0.008	0.272
alpha-blockers	Fracture	IVW	12.14	11	0.353			
ACEIs	Fracture	MR Egger	2.57	3	0.462	-0.062	0.012	0.488
ACEIs	Fracture	IVW	3.05	4	0.549			
ARBs	Fracture	MR Egger	0.11	2	0.947	0.151	0.011	0.137
ARBs	Fracture	IVW	2.32	3	0.509			
BBs	Fracture	MR Egger	18.72	18	0.409	0.004	0.005	0.999
BBs	Fracture	IVW	18.72	19	0.475			
CCBs	Fracture	MR Egger	67.68	62	0.289	-0.026	0.003	0.157
CCBs	Fracture	IVW	69.87	63	0.258			
loop diuretics	Fracture	MR Egger	8.95	7	0.256	-0.050	0.009	0.310
loop diuretics	Fracture	IVW	10.27	8	0.247			
PSDs	Fracture	IVW	0.80	1	0.372			
thiazide diuretics	Fracture	MR Egger	0.57	2	0.751	0.174	0.023	0.312
thiazide diuretics	Fracture	IVW	1.60	3	0.660			
alpha-blockers	eBMD	MR Egger	15.22	8	0.055	0.004	0.003	0.837
alpha-blockers	eBMD	IVW	15.30	9	0.083			
ACEIs	eBMD	MR Egger	4.18	2	0.124	0.026	0.005	0.247
ACEIs	eBMD	IVW	6.99	3	0.072			
ARBs	eBMD	IVW	0.21	1	0.646			
BBs	eBMD	MR Egger	7.43	9	0.590	0.006	0.002	0.129
BBs	eBMD	IVW	9.73	10	0.460			
CCBs	eBMD	MR Egger	108.39	53	0.000	-0.004	0.001	0.258
CCBs	eBMD	IVW	111.00	54	0.000			
loop diuretics	eBMD	MR Egger	41.85	6	0.000	-0.031	0.007	0.178
loop diuretics	eBMD	IVW	54.49	7	0.000			
PSDs	eBMD	IVW	6.13	1	0.013			
thiazide diuretics	eBMD	MR Egger	2.66	1	0.103	-0.006	0.012	0.822
thiazide diuretics	eBMD	IVW	2.79	2	0.247			
alpha-blockers	TB-BMD	MR Egger	10.91	10	0.364	-0.009	0.007	0.217
alpha-blockers	TB-BMD	IVW	12.57	11	0.322			
ACEIs	TB-BMD	MR Egger	0.97	3	0.808	-0.010	0.010	0.341
ACEIs	TB-BMD	IVW	1.88	4	0.758			
ARBs	TB-BMD	MR Egger	0.02	1	0.880	0.006	0.010	0.514
ARBs	TB-BMD	IVW	0.45	2	0.799			
BBs	TB-BMD	MR Egger	9.11	18	0.957	0.003	0.005	0.449
BBs	TB-BMD	IVW	9.68	19	0.960			
CCBs	TB-BMD	MR Egger	73.76	62	0.146	-0.001	0.002	0.513
CCBs	TB-BMD	IVW	74.35	63	0.155			
loop diuretics	TB-BMD	MR Egger	6.50	7	0.482	-0.015	0.007	0.034
loop diuretics	TB-BMD	IVW	10.99	8	0.202			
PSDs	TB-BMD	IVW	0.01	1	0.926			
thiazide diuretics	TB-BMD	MR Egger	3.69	2	0.158	-0.001	0.029	0.978
thiazide diuretics	TB-BMD	IVW	3.69	3	0.296			

ACEIs, angiotensin-converting enzyme inhibitors; ARBs, angiotensin receptor blockers; BBs, beta-blockers; CCBs, calcium channel blockers; BBs, beta-blockers; PSDs, potassium sparing diuretics; eBMD, estimated heel bone mineral density; TB-BMD, total body bone mineral density; IVW: inverse-variance weighted; MR, Mendelian randomization.

Supplementary Table 4. Associations of genetically predicted antihypertensive drugs with fracture

Exposure	Outcome	Method	OR (95%CI)	*P*	*P*-adjusted
alpha-blockers	Fracture	IVW	1.075 (0.946,1.223)	0.268	0.429
		Weighted median	1.092 (0.927,1.288)	0.293	
		MR-Lasso	1.075 (0.946,1.223)	0.268	
		MR-Robust	1.061 (0.956,1.177)	0.269	
		MRRAPS	1.064 (0.933,1.212)	0.356	
		MRPRESSO	1.075 (0.946,1.223)	0.292	
ACEIs	Fracture	IVW	1.158 (0.987,1.359)	0.071	0.190
		Weighted median	1.188 (0.979,1.440)	0.080	
		MR-Lasso	1.158 (0.987,1.359)	0.071	
		MR-Robust	1.159 (1.020,1.317)	0.024	
		MRRAPS	1.161 (0.978,1.378)	0.087	
		MRPRESSO	1.158 (1.008,1.332)	0.108	
ARBs	Fracture	IVW	0.671 (0.538,0.838)	4.42E-04	0.004
		Weighted median	0.690 (0.515,0.924)	1.26E-02	
		MR-Lasso	0.671 (0.538,0.838)	4.42E-04	
		MR-Robust	0.673 (0.559,0.812)	3.33E-05	
		MRRAPS	0.666 (0.514,0.862)	1.99E-03	
		MRPRESSO	0.671 (0.552,0.816)	2.80E-02	
BBs	Fracture	IVW	0.982 (0.908,1.063)	0.657	0.735
		Weighted median	0.996 (0.892,1.113)	0.947	
		MR-Lasso	0.982 (0.908,1.063)	0.657	
		MR-Robust	0.973 (0.904,1.049)	0.477	
		MRRAPS	0.973 (0.895,1.057)	0.513	
		MRPRESSO	0.982 (0.908,1.062)	0.660	
CCBs	Fracture	IVW	1.072 (1.026,1.121)	2.11E-03	0.013
		Weighted median	1.088 (1.014,1.168)	1.90E-02	
		MR-Lasso	1.084 (1.038,1.132)	2.56E-04	
		MR-Robust	1.081 (1.036,1.129)	3.59E-04	
		MRRAPS	1.083 (1.033,1.136)	1.03E-03	
		MRPRESSO	1.072 (1.026,1.121)	3.11E-03	
loop diuretics	Fracture	IVW	1.087 (0.940,1.258)	0.260	0.429
		Weighted median	1.147 (0.963,1.367)	0.125	
		MR-Lasso	1.087 (0.940,1.258)	0.260	
		MR-Robust	1.097 (0.954,1.262)	0.193	
		MRRAPS	1.109 (0.955,1.287)	0.175	
		MRPRESSO	1.087 (0.940,1.258)	0.292	
PSDs	Fracture	IVW	1.233 (0.932,1.629)	0.142	0.310
		MR-Robust	1.234 (1.040,1.464)	0.016	
		MRRAPS	1.237 (0.908,1.684)	0.178	
		IVW	0.822 (0.651,1.038)	0.099	
thiazide diuretics	Fracture	Weighted median	0.851 (0.642,1.128)	0.262	0.238
		MR-Lasso	0.822 (0.651,1.038)	0.099	
		MR-Robust	0.827 (0.664,1.030)	0.090	
		MRRAPS	0.819 (0.631,1.062)	0.131	
		MRPRESSO	0.822 (0.693,0.974)	0.109	

ACEIs, angiotensin-converting enzyme inhibitors; ARBs, angiotensin receptor blockers; BBs, beta-blockers; CCBs, calcium channel blockers; PSDs, potassium sparing diuretics; IVW inverse-variance weighted; MR, Mendelian randomization; MR-RAPS, Robust Adjusted Profile Score; MR-PRESSO, Mendelian Randomization Pleiotropy RESidual Sum and Outlier.

Supplementary Table 5. Associations of genetically predicted antihypertensive drugs with TB-BMD

Exposure	Outcome	Method	β (95%CI)	*P*	*P*-adjusted
alpha-blockers	TB-BMD	IVW	-0.075 (-0.190,0.040)	0.200	0.394
		Weighted median	-0.052 (-0.200,0.096)	0.490	
		MR-Lasso	-0.075 (-0.190,0.040)	0.200	
		MR-Robust	-0.071 (-0.185,0.043)	0.221	
		MRRAPS	-0.074 (-0.193,0.045)	0.222	
		MRPRESSO	-0.075 (-0.190,0.040)	0.226	
ACEIs	TB-BMD	IVW	0.003 (-0.138,0.144)	0.969	0.969
		Weighted median	0.026 (-0.139,0.191)	0.759	
		MR-Lasso	0.003 (-0.138,0.144)	0.969	
		MR-Robust	0.006 (-0.082,0.094)	0.888	
		MRRAPS	0.003 (-0.148,0.153)	0.970	
		MRPRESSO	0.003 (-0.094,0.100)	0.957	
ARBs	TB-BMD	IVW	0.358 (0.109,0.607)	4.90E-03	0.022
		Weighted median	0.358 (0.040,0.675)	2.74E-02	
		MR-Lasso	0.358 (0.109,0.607)	4.90E-03	
		MR-Robust	0.358 (0.199,0.517)	1.06E-05	
		MRRAPS	0.360 (0.064,0.656)	1.70E-02	
beta-blockers	TB-BMD	IVW	-0.015 (-0.084,0.054)	0.674	0.735
		Weighted median	-0.025 (-0.117,0.068)	0.600	
		MR-Lasso	-0.015 (-0.084,0.054)	0.674	
		MR-Robust	-0.022 (-0.091,0.047)	0.536	
		MRRAPS	-0.018 (-0.091,0.055)	0.625	
		MRPRESSO	-0.015 (-0.064,0.034)	0.562	
CCBs	TB-BMD	IVW	0.038 (-0.001,0.076)	0.056	0.169
		Weighted median	0.033 (-0.028,0.095)	0.288	
		MR-Lasso	0.038 (-0.001,0.076)	0.056	
		MR-Robust	0.037 (0.003,0.071)	0.031	
		MRRAPS	0.038 (-0.002,0.079)	0.065	
		MRPRESSO	0.038 (-0.001,0.076)	0.059	
loop diuretics	TB-BMD	IVW	0.068 (-0.065,0.202)	0.316	0.449
		Weighted median	0.157 (0.004,0.311)	0.044	
		MR-Lasso	0.068 (-0.065,0.202)	0.316	
		MR-Robust	0.077 (-0.052,0.207)	0.243	
		MRRAPS	0.086 (-0.045,0.218)	0.199	
		MRPRESSO	0.068 (-0.065,0.202)	0.345	
PSDs	TB-BMD	IVW	-0.609 (-0.885, -0.333)	1.55E-05	1.86E-04
		MR-Lasso	-0.609 (-0.885, -0.333)	1.55E-05	
		MR-Robust	-0.609 (-0.729, -0.488)	3.93E-23	
		MRRAPS	-0.608 (-0.959, -0.258)	6.70E-04	
thiazides diuretics	TB-BMD	IVW	0.240 (0.012,0.467)	0.039	0.134
		Weighted median	0.239 (-0.036,0.513)	0.088	
		MR-Lasso	0.240 (0.012,0.467)	0.039	
		MR-Robust	0.237 (-0.001,0.475)	0.051	
		MRRAPS	0.248 (0.013,0.482)	0.039	

	MRPRESSO	0.240 (0.012,0.467)	0.131

ACEIs, angiotensin-converting enzyme inhibitors; ARBs, angiotensin receptor blockers; BBs, beta-blockers; CCBs, calcium channel blockers; BBs, beta-blockers; PSDs, potassium sparing diuretics; IVW: inverse-variance weighted; MR, Mendelian randomization; MR-RAPS, Robust Adjusted Profile Score; MR-PRESSO, Mendelian Randomization Pleiotropy RESidual Sum and Outlier.; TB-BMD, total body bone mineral density.

Supplementary Table 6. Associations of genetically predicted antihypertensive drugs with eBMD

Exposure	Outcome	Method	β (95%CI)	*P*	*P*-adjusted
alpha-blockers	eBMD	IVW	-0.030 (-0.088,0.028)	0.318	0.449
		Weighted median	-0.043 (-0.097,0.011)	0.122	
		MR-Robust	-0.029 (-0.088,0.030)	0.337	
		MRRAPS	-0.025 (-0.087,0.037)	0.431	
		MRPRESSO	-0.030 (-0.088,0.028)	0.339	
ACEIs	eBMD	IVW	-0.038 (-0.117,0.040)	0.341	0.454
		Weighted median	-0.007 (-0.067,0.052)	0.816	
		MR-Lasso	-0.005 (-0.062,0.053)	0.871	
		MR-Robust	-0.037 (-0.105,0.031)	0.285	
		MRRAPS	-0.053 (-0.134,0.029)	0.204	
		MRPRESSO	-0.038 (-0.117,0.040)	0.395	
ARBs	eBMD	IVW	0.295 (0.212,0.378)	2.73E-12	6.55E-11
		MR-Robust	0.295 (0.256,0.334)	2.65E-50	
		MRRAPS	0.296 (0.172,0.420)	2.97E-06	
BBs	eBMD	IVW	0.041 (0.010,0.072)	4.09E-02	0.501
		Weighted median	0.024 (-0.019,0.067)	2.42E-02	
		MR-Lasso	0.041 (0.010,0.072)	4.09E-02	
		MR-Robust	0.038 (0.005,0.072)	3.82E-02	
		MRRAPS	0.040 (0.007,0.073)	4.01E-02	
		MRPRESSO	0.041 (0.010,0.072)	4.09E-02	
CCBs	eBMD	IVW	-0.007 (-0.029,0.014)	0.502	0.602
		Weighted median	0.010 (-0.010,0.030)	0.317	
		MR-Lasso	0.003 (-0.011,0.017)	0.689	
		MR-Robust	0.001 (-0.018,0.021)	0.886	
		MRRAPS	-0.012 (-0.041,0.017)	0.429	
		MRPRESSO	-0.007 (-0.029,0.014)	0.504	
loop diuretics	eBMD	IVW	-0.010 (-0.115,0.094)	0.846	0.883
		Weighted median	0.011 (-0.046,0.068)	0.698	
		MR-Lasso	0.028 (-0.063,0.119)	0.550	
		MR-Robust	-0.007 (-0.100,0.086)	0.881	
		MRRAPS	0.043 (-0.051,0.138)	0.372	
		MRPRESSO	-0.010 (-0.115,0.094)	0.852	
PSDs	eBMD	IVW	-0.050 (-0.128,0.029)	0.214	0.394
		MR-Robust	-0.050 (-0.183,0.083)	0.459	
		MRRAPS	-0.021 (-0.196,0.155)	0.816	
thiazide diuretics	eBMD	IVW	0.106 (0.031,0.182)	5.59E-03	0.022
		Weighted median	0.108 (0.008,0.207)	3.42E-02	
		MR-Lasso	0.106 (0.017,0.195)	1.90E-02	
		MR-Robust	0.107 (0.029,0.185)	7.22E-03	
		MRRAPS	0.110 (0.024,0.197)	1.24E-02	

ACEIs, angiotensin-converting enzyme inhibitors; ARBs, angiotensin receptor blockers; BBs, beta-blockers; CCBs, calcium channel blockers; PSDs, potassium sparing diuretics; IVW, inverse-variance weighted; MR, Mendelian randomization; MR-RAPS, Robust Adjusted Profile Score; MR-PRESSO, Mendelian Randomization Pleiotropy RESidual Sum and Outlier.; BMD, bone mineral density; BMD, bone mineral density; eBMD, estimated heel BMD.

Supplementary Table 7. SNPs associated with risk factors of osteoporosis identified with PhenoScanner

SNP	Trait	*P*	Study	PMID
rs3821843	BMI	4.77E-06	Neale B	UKBB
rs79480822	Alzheimer disease and age of onset	7.00E-07	Herold C	26830138
rs1862341	Smoking status: current	7.92E-07	Neale B	UKBB
rs10828399	BMI	4.85E-09	Akiyama M	28892062
rs2612015	BMI in females greater than 50 years of age	2.70E-06	GIANT	26426971
rs72786098	Small vessel stroke	2.00E-08	Cheng YC	26732560

BMI, Body mass index

Supplementary Table 8. The associations between genetic proxies for antihypertensive drugs and fracture, after excluding pleiotropic SNPs

Exposure	Outcome	Method	OR (95%CI)	*P*
alpha-blockers	Fracture	IVW	1.075 (0.946,1.223)	0.268
ACEIs	Fracture	IVW	1.128 (0.952,1.337)	0.165
ARBs	Fracture	IVW	0.671 (0.538,0.838)	4.42E-4
BBs	Fracture	IVW	0.989 (0.913,1.072)	0.791
CCBs	Fracture	IVW	1.072 (1.024,1.121)	2.77E-3
loop diuretics	Fracture	IVW	1.148 (1.000,1.318)	0.050
PSDs	Fracture	IVW	1.233 (0.932,1.629)	0.142
thiazides diuretics	Fracture	IVW	0.822 (0.651,1.038)	0.099

ACEIs, angiotensin-converting enzyme inhibitors; ARBs, angiotensin receptor blockers; BBs, beta-blockers; CCBs, calcium channel blockers; BBs, beta-blockers; PSDs, potassium sparing diuretics; IVW: inverse-variance weighted.

Supplementary Table 9. The association between genetic proxies for antihypertensive drugs and BMD, after excluding pleiotropic SNPs

Exposure	Outcome	Method	β (95%CI)	*P*
alpha-blockers	TB-BMD	IVW	-0.075 (-0.19,0.040)	0.200
ACEIs	TB-BMD	IVW	0.013 (-0.135,0.160)	0.865
ARBs	TB-BMD	IVW	0.358 (0.109,0.607)	0.005
BBs	TB-BMD	IVW	-0.016 (-0.086,0.054)	0.651
CCBs	TB-BMD	IVW	0.052 (0.010,0.094)	0.015
loop diuretics	TB-BMD	IVW	0.068 (-0.065,0.202)	0.316
PSDs	TB-BMD	IVW	-0.609 (-0.885, -0.333)	1.55E-05
thiazides diuretics	TB-BMD	IVW	0.240 (0.012,0.467)	0.039
alpha-blockers	eBMD	IVW	-0.015 (-0.068,0.038)	0.590
ACEIs	eBMD	IVW	-0.042 (-0.138,0.054)	0.392
ARBs	eBMD	IVW	0.295 (0.212,0.378)	2.73E-12
BBs	eBMD	IVW	0.041 (0.010,0.072)	4.09E-02
CCBs	eBMD	IVW	-0.002 (-0.018,0.014)	0.842
loop diuretics	eBMD	IVW	0.028 (-0.063,0.119)	0.550
PSDs	eBMD	IVW	-0.050 (-0.128,0.029)	0.214
thiazides diuretics	eBMD	IVW	0.082 (0.002,0.162)	0.044
alpha-blockers	eBMD	IVW	-0.015 (-0.068,0.038)	0.590

ACEIs, angiotensin-converting enzyme inhibitors; ARBs, angiotensin receptor blockers; BBs, beta-blockers; CCBs, calcium channel blockers; BBs, beta-blockers; PSDs, potassium sparing diuretics; eBMD, estimated heel bone mineral density; TB-BMD, total body bone mineral density; IVW: inverse-variance weighted; MR, Mendelian randomization.
